# Excitation-Inhibition Imbalance in Migraine: From Neurotransmitters to Brain Oscillations

**DOI:** 10.3390/ijms241210093

**Published:** 2023-06-13

**Authors:** Louise O’Hare, Luca Tarasi, Jordi M. Asher, Paul B. Hibbard, Vincenzo Romei

**Affiliations:** 1 Division of Psychology, Nottingham Trent University, Nottingham NG1 4FQ, UK; 2 Centro Studi e Ricerche in Neuroscienze Cognitive, Dipartimento di Psicologia, Alma Mater Studiorum—Università di Bologna, Campus di Cesena, Via Rasi e Spinelli, 176, 47521 Cesena, Italy; luca.tarasi2@unibo.it; 3 Department of Psychology, University of Essex, Colchester CO4 3SQ, UK; jashera@essex.ac.uk (J.M.A.); phibbard@essex.ac.uk (P.B.H.); 4 Facultad de Lenguas y Educación, Universidad Antonio de Nebrija, 28015 Madrid, Spain

**Keywords:** migraine, dysrhythmia, alpha, gamma, glutamate, GABA, computational models, excitation–inhibition imbalance, homeostatic mechanism

## Abstract

Migraine is among the most common and debilitating neurological disorders typically affecting people of working age. It is characterised by a unilateral, pulsating headache often associated with severe pain. Despite the intensive research, there is still little understanding of the pathophysiology of migraine. At the electrophysiological level, altered oscillatory parameters have been reported within the alpha and gamma bands. At the molecular level, altered glutamate and GABA concentrations have been reported. However, there has been little cross-talk between these lines of research. Thus, the relationship between oscillatory activity and neurotransmitter concentrations remains to be empirically traced. Importantly, how these indices link back to altered sensory processing has to be clearly established as yet. Accordingly, pharmacologic treatments have been mostly symptom-based, and yet sometimes proving ineffective in resolving pain or related issues. This review provides an integrative theoretical framework of excitation–inhibition imbalance for the understanding of current evidence and to address outstanding questions concerning the pathophysiology of migraine. We propose the use of computational modelling for the rigorous formulation of testable hypotheses on mechanisms of homeostatic imbalance and for the development of mechanism-based pharmacological treatments and neurostimulation interventions.

## 1. Introduction

### 1.1. Background

Migraine is a common and debilitating neurological disorder, affecting around 10% of the population [1] and having a substantial social and economic burden [2]. Migraine is characterised by a headache lasting 4–72 h when untreated, which tends to be unilateral, pulsating, and of moderate to severe pain intensity [3]. Migraine typically affects people of working age, with the most prevalent age group being 30–39 years [4], onset typically after the age of 12 and the number of cases declining after the age of 40 [5], although paediatric migraine and migraine persisting into later life are also common. Migraine does not necessarily last for the entire lifetime; a follow-up study showed that after 12 years, around 42% went into remission. However, the same study showed that 12% experienced progression (deterioration) of the disorder [6].

Episodic migraine has two main subtypes, migraine with (MA) and without (MO) aura, although there are several other types, including chronic migraine (more than 15 attacks per month), menstrual migraine (characterised as following the menstrual cycle), and familial hemiplegic migraine (characterised by fully reversible motor weakness during the aura stage and a family history of the disorder) [7]. The migraine aura is most commonly visual in nature, although it can also manifest in other sensory modalities. The aura is typically characterised by a shimmering zig-zag pattern around a central scotoma (blind patch of the visual field) that starts small and progresses to engulf more of the visual field over a time period of around 5–60 min [8]. The progression of the visual aura has been linked to a phenomenon called cortical spreading depolarisation and depression (CSD) [9]. This is a spreading wave of very strong cortical activity (depolarisation), followed by a cortical silent period (depression), thought to manifest as hallucinations and scotoma, respectively. Although there is fMRI evidence to support this view [10], it has been challenged [11] for several reasons. For example, CSD has never been recorded in human migraine and cannot account for attacks without aura. Additionally, a fundamental concept is that the pain of migraine is due to vasodilation caused by the large demand for neural resources during the aura. However, the time course of vasodilation is incompatible with the time course of the pain experienced in migraine (see [12] for discussion).

The migraine cycle consists firstly of the headache attack itself (ictal stage). This is preceded by a premonitory stage, lasting from hours to days (preictal stage). Symptoms during this phase include tiredness, cravings, and light and sound sensitivity. After the attack, the individual may also feel unwell for up to several days; again, this can include tiredness and difficulty concentrating [13].

#### 1.1.1. Migraine Pathophysiology Is Unclear

Migraine is a sensory disorder [14] with strong links to visual processing in particular. Firstly, experiencing photophobia (aversion to light) during the attack is one of the diagnostic criteria [3]. Secondly, people with migraine report aversion to visual stimuli such as bright lights [15] and striped patterns [16] in between attacks. Visual stimuli can also trigger migraine attacks [17].

Migraine has a strong genetic component, and there is increasing evidence of an inherited mutation in familial hemiplegic migraine [18]. Migraine tends to change over the lifetime-there is some evidence of both remission and progression. Over the course of a year, around 84% will be stable, and 3% will progress to chronic migraine [19]. In adults between 25 and 64, there was remission for 42% but progression for 20% after 12 years [6]. Remission is relatively common in childhood migraine, with 27% being migraine free before the age of 25 [20].

There are some important differences between episodic and chronic migraine, although the distinction is based on clinical characteristics (more than 15 migraine attacks per month [3]). A review of the literature showed that those with chronic migraine typically show impaired function in the periaqueductal grey, lesions in white matter, and an increased association with cardiovascular disorder [19]. This suggests there are structural changes in chronic migraine; however, it has yet to be established whether these changes are the cause or the result of migraine. It has been suggested that repeated migraine attacks may cause damage to the brain [21].

In a systematic review, Buse et al. [22] showed the risk factors for progression from episodic to chronic migraine include several clinical features of the headache itself, including headache frequency, migraine-related nausea, and age of onset. In paediatric migraine, the age of onset of migraine is also important for the progression of the disorder. Those with onset before the age of 6 were 4.2 times more likely to get worse compared to migraine starting later in childhood [23]. This suggests a possible predisposition to more severe migraine that manifests more quickly compared to others.

Given the purported genetic nature, the heterogeneity of phenotypal characterisations and its diverse temporal progressions, several hypotheses have been formulated to better understand the aetiology and pathophysiology of migraine [14]. In particular, research has delved into different levels of investigation ranging from molecular (e.g., [24] to neurophysiological mechanisms, including oscillatory brain dynamics [25], that may lead to the insurgence, maintenance and progression towards chronification versus resolution of migraine [26].

While there have been several suggestions put forward, a common working hypothesis has posited an excessive cortical excitation [27]. As a counterpart, there have also been suggestions of a lack of inhibitory processes [25].

#### 1.1.2. Goals of the Review

Accurate, multilevel scrutiny of the potential of an imbalance of excitation and inhibition is the focus and primary goal of this review. In reviewing the empirical details, we note that the evidence collected so far, albeit persuasive, is often patchy and partially contradictory. Moreover, despite intense investigation, there is largely a lack of cross-talk between the molecular, cellular, and neurophysiological research approaches. Thus, a second goal of the current review is an attempt to bridge the gap between different research approaches for a more coherent understanding of the pathophysiology of migraine. Given the level of complexity introduced by the interaction of these different levels of analyses, mathematical modelling will be introduced as a valuable tool for making testable hypotheses.

**Figure 1:**
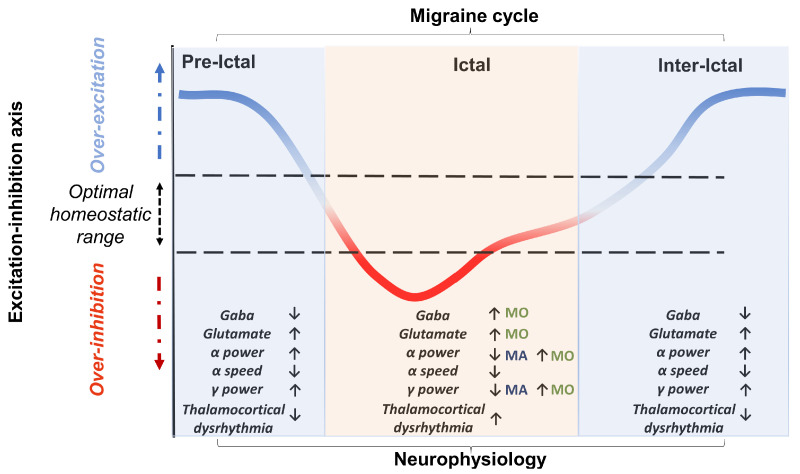
The proposed perspective view considers the imbalance between excitation–inhibition characterising migraine as a dynamic attempt to reach an optimal homeostatic balance. This attempt to stay in the optimal excitability range is characterised by specific and different electroencephalographic and neutrotransmitter markers across the migraine cycle. The orientation of the arrows indicates the progression of the marker throughout the migraine cycle. In particular, in the migraine phases characterised by overexcitation (preictal and interictal), a trend consisting of a decrease, compared to the ictal phases in both GABA concentration and dysrhythmia between the thalamus and cortex, can be observed. In contrast, alpha and gamma power increase in these phases. In the ictal phases, a heightened dysrhythmia in the thalamocortical activity can be detected. Moreover, in this phase, the trajectory of the neurophysiological marker varies according to the type of migraine (MO vs MA). In MO, there is an increase in GABA concentration, but alpha and gamma power remains higher. In contrast, in MA, there is a desynchronisation in both alpha and gamma frequency bands. Two other parameters noted to be abnormal in migraineurs are glutamate concentrations (increased) and alpha speed (reduced). However, these two markers do not appear to be significantly modulated by the migraine cycle and thus they could be considered as phase-non-specific markers of migraine. It is important to emphasise that the results in the literature are mixed and patchy. Therefore, future follow-up studies will be needed to empirically assess the precise correspondence between migraine phase and directionality of the effect on physiological parameters.

A third goal of the review will be to propose a perspective in an attempt to resolve the often-conflicting findings by treating the excitation–inhibition imbalance hypothesis as a dynamic but failing attempt to maintain a homeostatic balance. This may thus range from periods of hyper-excitability (preceding the ictal and preictal stages) to refractory periods of hypo-excitability (preictal periods with or without aura and ictal stage) (see Figure 1). Moreover, such an altered homeostatic process may change as the migraine progresses, towards a resolution or chronification.

The final section of the review will be dedicated to current perspectives in the treatment of migraine moving the focus from symptomatology-based treatment to mechanism-based interventions and ranging from pharmacologic to neurostimulation approaches.

We will introduce these arguments by first looking at the hypothesis of the imbalance of excitation and inhibition as indexed by neural oscillations. There is ample evidence suggesting that neural oscillations are disordered in migraine. In particular, thalamocortical dysrhythmia has been suggested as a possible mechanism [28] accounting for the excitation–inhibition imbalance hypothesis. Specifically, alpha-band oscillations (7–13 Hz) are thought to have an active role in controlling the excitability of the cortex to incoming sensory stimuli in a periodic fashion, and these oscillations also seem to control the gamma-band oscillations (>35 Hz) that seem to be related to the coding of stimulus properties [29]. The central idea of thalamocortical dysrhythmia is that the alpha- and gamma-band oscillations are imbalanced, and this is the result of disordered signalling between the thalamus and the cortex. The review will explore the empirical and theoretical evidence supporting the role that altered alpha and gamma-band oscillations, as well as their interactions, may account for altered signalling of sensory information.

Importantly, the review will attempt to link this first level of evidence to disorders of neurotransmitters. Indeed, the excitation–inhibition imbalance, which may be observed as an alteration of oscillatory properties as introduced above, might be due to disordered neurotransmitter levels. For example, an imbalance of GABA and glutamate synthesis, reuptake and metabolism is linked to seizures [30]. This is important because there are several similarities between migraine and epilepsy [31], although it must be noted that these are distinct disorders. In this review, we will explore the evidence suggesting that there may be excessive glutamate but reduced GABA levels in migraine and how this might relate to disordered neural oscillations. It has been suggested that differences in neurotransmitter levels in migraine may be homeostatic [32]. GABA and glutamate levels show fluctuation in the preictal, ictal, and postictal stages, which could be evidence of homeostatic compensation.

If an imbalance in inhibition and excitation is either a correlate or a result of the disorder, then the changes in oscillations and neurotransmitter levels should relate to changes in clinical characteristics over the course of the disorder, such as disease duration and progression. If these processes are in fact linked to the origins of the disorder, then these differences should be seen in the initial stages or in childhood manifestations.

Finally, we will explore therapies for redressing the excitation–inhibition imbalance in migraine, particularly focusing on non-medication interventions. New approaches offer promising avenues for the treatment of migraine for several reasons. However, for many, there is dissatisfaction with current medication, and many individuals are contra-indicated for several medications. Therefore, it may be possible to develop alternative interventions addressing neurochemical imbalances and disordered oscillatory activity through vitamin supplementation and neurostimulation, respectively.

## 2. Differences in Alpha-Band Oscillations

### 2.1. Introduction to Alpha

Alpha-band oscillations are the most prominent and easy-to-identify brain rhythm in the human power spectrum, spanning between 7 and 13 Hz and peaking in the parieto-occipital regions [33]. This neural feature has been associated with a large range of cognitive processes such as attention, perception, memory, and predictive processing [34,35,36,37,38,39].

The pattern of oscillatory synchronisation and amplitude is known to fluctuate based on an observer’s state of vigilance. For example, when an observer is in a restful state (i.e., eyes closed), alpha-band oscillations synchronise, increasing their amplitude [40]. In contrast, when in a state of readiness immediately preceding stimulus onset, alpha-band oscillations desynchronise, reducing their amplitude. Furthermore, alpha rhythms have a fundamental role in maintaining an active and flexible mechanism of inhibition that reduces the processing capabilities of a given area of the brain to task-irrelevant stimuli [41]. According to this framework, cortical excitability is negatively correlated with the amplitude of alpha oscillations. Typically, the role played by alpha rhythms in regulating cortical excitability is assumed to be a determining factor for accurate stimulus perception. For example, Van Dijk et al. [42] demonstrated prestimulus alpha desynchronisation was greater for correctly identified stimuli (hits) than for incorrectly identified stimuli (misses). However, recent findings have cast out this classical interpretation, demonstrating that alpha desynchronisation is connected to an increased likelihood of reporting the presence of targets in visual tasks, regardless of its actual presence [43,44]. Therefore, the increase in cortical excitability due to alpha desynchronisation would not sharpen the sensory response but amplify it by affecting signal and noise processing equally.

### 2.2. Differences in Alpha during Attacks

Since alpha plays a role in defining the level of excitation–inhibition of cortical areas, several lines of research assume that dysregulation of the alpha band may promote the onset of migraine. However, it is still unclear whether the mechanism that elicits migraine is due to inflexible excitation or inhibition of cortical activity. Using MEG, Hall et al. [45] showed that, for the first 5 min of the aura, there is strong desynchronisation of occipital alpha-band activity that returns to normal over the following 10 min. This desynchronisation would testify to an increased excitability of the visual areas. However, Bjørk and Sand [46] and Bjørk et al. [47] showed evidence of increased occipital alpha power during the attack phase. Both of these were predominantly MO participants, suggesting perhaps the reduced alpha power found during the aura phase is related to the hallucinations themselves. Moreover, during the migraine attack, MO patients showed decreased alpha power and coherence compared to controls in fronto-central and posterior networks, which normalised before and after the attack phase itself [48]. This suggests that differences in findings could be due to the specific stage of the migraine cycle at the time of testing.

### 2.3. Differences in Alpha between Attacks

There are also differences in alpha-band oscillations between attacks. There is evidence to suggest a trend for increased resting-state occipital alpha power over several cortical regions in MA compared to controls [49] and greater occipital alpha power in MA compared to MO [50]. There is evidence of increased occipital resting-state alpha power in a mixed migraine group compared to the control group [51] and higher power in the lower alpha range (8–10 Hz) over occipital areas in a mixed migraine group compared to a control group [52].

As well as differences in power, part of the scientific literature has focused on the role that individual alpha frequency may play in the development of migraine. Individual alpha frequency is a stable and heritable neurophysiological marker [53,54] able to predict individual differences in several cognitive domains. For example, individual alpha frequency correlates with memory task performance [55], with the g factor of intelligence [56] and with attentional performance [57]. Moreover, faster alpha oscillations lead to higher temporal resolution [58] and more accurate detection performance [59]. Furthermore, reduction (or acceleration) of occipital alpha speed by means of tACS widens (or shrinks) the sensory integration window, thus increasing (or reducing) the proneness to experience the double-flash illusion [60]. Neufeld et al. [61] found faster peak alpha frequency over posterior areas in migraine compared to the control groups between attacks. However, Bjørk et al. [47] did not find a difference in any metric of occipital alpha (peak frequency or power) for controls compared to migraine in between attacks, although these authors and others [47,62] did find occipital peak alpha frequency was negatively related to disease duration for a mixed MO and MA migraine group; those who experienced migraine for longer across the lifetime had a slower alpha-band peak frequency. This is potentially clinically important as there is a relationship between those with slower peak alpha frequency at rest being more sensitive to painful stimuli [63] (but see [64]). There is also a developmental trajectory in the peak alpha frequency, which speeds up from childhood to adulthood and could result from the strengthening of thalamocortical connections [65]. Converging evidence assumes that alpha-band oscillations are synchronised between the thalamus and the visual areas (for a review, see [66]). Specifically, thalamocortical activity is synchronised with the peaks and troughs of alpha-band oscillations [67]. Crucially, during a migraine attack, the peak alpha frequency shows greater variability [68], which could result from an instability in the thalamic generators of alpha-band oscillations.

### 2.4. Differences in Alpha during Visual Stimulation

There is also evidence for differences in the synchronization of activity in the alpha band between a mixed migraine group (MO and MA) and controls during visual stimulation. Fong et al. [69] demonstrated that migraineurs had significantly less posterior alpha power prior to the onset of the stimulus relative to controls. Moreover, migraineurs had significantly greater poststimulus alpha desynchronisation. These findings suggest the presence of hyperresponsiveness in the visual area of migraineurs. However, there are several studies that point in the opposite direction. Angelini et al. [70] used flashing light of a range of frequencies, between 3 and 24 Hz and found increased synchronisation in MO compared to controls over the whole head. Other authors have replicated this finding of increased alpha synchronisation over the whole of the cortex in migraine compared to control using flash stimulation [71,72]. In a further study, this increased synchronisation in MO was found predominantly in the occipital cortex and was reduced with levetiracetam [73], but unaffected with 1 Hz TMS [74]. Levetiracetam is an antiepileptic medication, thought to strengthen inhibition by blockading P/Q-type Ca^2+^ channels and delayed K+ exchange; additionally, levetiracetam has effects on GABA_A_ receptors, but only in epileptic tissue [75]. Additionally, 1 Hz TMS is thought to have inhibitory effects [76]; however, this view has been challenged [77]. There is evidence to suggest that in MA, 1 Hz TMS applied to the occipital areas may actually have the effect of increasing excitation [78]. Therefore, medications to increase inhibition seem to reverse the increased alpha synchronisation in migraine, but direct stimulation from TMS has no effect. This could indicate that TMS is not having the desired effect of increasing inhibition.

### 2.5. Differences in Alpha between Migraine Subtypes

Importantly, there was increased alpha-band phase synchronisation between occipital, parietal, central, and frontal channels in MO but not in MA during steady-state visual evoked potential (SSVEP) flash stimulation compared to resting activity at a range of frequencies (9, 18, 21, 24, 27 Hz) [79]. However, there is evidence of less spatially coherent alpha-band activity over frontal clusters both during visual and auditory tasks and at rest in a predominantly MA group [80], which is the opposite finding to the work of De Tommaso et al. [79] in a predominantly MO group. This is important as it shows there might be differences between the mechanisms associated with MO and MA. Moreover, there is increased alpha power in MA compared to other neurological disorders, which is helpful for discrimination between them.

### 2.6. Summary

There is a complex pattern of results regarding alpha-band oscillations in migraine, as summarised in Table 1. These oscillations appear to be slower, higher in power, and more variable in those with migraine compared to controls, although not all studies are in agreement with this result. This could be due to differing effects of the migraine cycle across studies. Even studies with interictal participants do not always have a clear process for measuring the proximity to the next migraine attack, and so instead of interictal migraine, it is possible they are in the preictal stages. In studies measuring alpha power in response to flickering stimuli, there are contradictory results. Some authors find decreased power, indicative of increased excitability, whilst others find increased alpha power in response to stimulation. This may be due to the subtype of migraine included in the study, as those experiencing MO specifically seem to show this increased synchronisation, e.g., [79], but not those experiencing MA [80]. Additionally, it must be noted that many individuals who experience migraine with aura attacks also experience attacks without aura [81]. Therefore, this can pose an issue for the studies reporting on ictal data, whether task performance or electrophysiological results, as the authors do not always report on what type of attack the migraine aura participants experienced. In the case of MO patients, it may be assumed that this is an attack without aura, but the same cannot necessarily be assumed for MA patients. Future studies investigating the effects of the migraine cycle in MA patients would be best advised to specify the type of attack when referring to the ictal period. Importantly, there are few studies using combined behavioural task and alpha oscillation measurements that have been used in control populations, e.g., [44]. Assessing both behavioural and clinical outcomes, using specific subgroups of migraine, and carefully estimating the point of the migraine cycle would be helpful to understand the role of alpha-band oscillations and perception in migraine.

## 3. Differences in Gamma-Band Oscillations

### 3.1. Introduction to Gamma

Gamma oscillations are high-frequency oscillations around 30–100 Hz, although this varies in different research papers [82]; therefore, efforts have been made here to specify the frequency that is referred to each time. Gamma-band oscillations have a strong genetic component [83], and there is increasing evidence that they are involved in a wide range of cognitive processes, including perception, attention, memory, and learning [84]. In particular, gamma oscillations have been linked to the binding of sensory information, allowing the brain to integrate information from different sensory modalities and create a coherent perceptual experience [85]. Moreover, recent research has also suggested that gamma oscillations may be disrupted in a variety of neuropsychiatric disorders, including schizophrenia, autism, and epilepsy [44,86,87]. It is important to note that there are three types of gamma-band oscillations commonly referred to as 40 Hz activity, steady-state, evoked, and induced [82]. Steady-state activity is the response to a repetitive stimulus, for example, flickering light. This can be estimated by spectral analysis of the time-series. Evoked activity is the response that is time-locked to a single stimulus and can be estimated by taking a grand average of the responses to the stimulus across trials and then conducting spectral analysis. Induced activity is activity that is related to the stimulus, but not time-locked to it, and is calculated from the time–frequency response (conducting spectral analysis and then averaging). Induced gamma-band (25–40 Hz) synchronisation (increased power) is thought to have a role in higher-order processing, such as the perception of coherent objects such as Gestalt figures [88]. Gamma-band oscillations in response to visual stimuli are relatively consistent for an individual over testing sessions [89]. However, gamma-band oscillations are in a broader bandwidth compared to alpha-band oscillations, and some individuals have two peaks, one around 40 Hz and another around 80 Hz [89].

### 3.2. The Origins of Gamma Oscillations

Gamma oscillations are thought to have two sources, thalamic and cortical, and the oscillations between these sources have been shown to be in phase [90], suggesting these are due to thalamocortical loops. However, this work was under anaesthesia [90], and by contrast, there was no evidence of phase synchronisation between the thalamic and cortical sources in response to visual stimulation. This indicates that these are independent sources of gamma-band oscillations [91]. Cortical gamma-band oscillations show a linear increase in frequency, but show a bell-shaped relationship (increasing then decreasing) with power with increasing velocity from high-contrast concentric circles. This nonlinear relationship is indicative of gain control [92]. Additionally, those with increased self-reported sensory sensitivity showed weaker suppression of occipital gamma-band oscillations in response to strong stimuli, suggesting a possible lack of gain control [93]. Therefore, gamma-band oscillations and their suppression with high-intensity stimuli might be of particular interest in migraine, as those with migraine typically report increased sensory sensitivity [16].

### 3.3. Gamma Activity during Attack

During the migraine aura, there is strong gamma-band desynchronisation (reduction in power) in temporal areas for the first minute of the migraine aura, slowly returning to baseline levels over a 16-min period [45]. However, Liu et al. [94] showed that there is an increase in gamma (50–90 Hz) power in the lateral cortical regions in those with acute migraine (both MA and MO) compared to age and gender-matched controls.

### 3.4. Gamma Activity in between Attacks

Resting-state gamma-band oscillations (30–90 Hz) in left frontal and temporal areas have higher power in migraine (MA and MO) compared to control groups, when accounting for correction for multiple comparisons (several frequency bands) [95]. Evoked gamma-band oscillation amplitude (20–35 Hz) recorded over the occipital region in response to visual stimulation is increased in between attacks in MA, but there was no difference in the latency of the wavelet peak, suggesting no difference in the peak frequency [28]. However, there was no difference between controls and MO groups in the gamma-band (70–90 Hz) response to painful laser stimulation on the hand and forehead, and moreover, the relationship between pain experience and central gamma-band oscillations only existed for the control, not the MO group [96], indicating that the experience of pain may be signalled differently in migraine compared to control groups. Using stimulation of the median nerve, Ren et al. [97] showed increased evoked gamma (30–80 Hz) band power in MO compared to controls over sensory regions. They also found evidence to suggest an increased functional pathway length for gamma oscillations that correlated with increased attack frequency. This increased functional pathway may mean that more brain regions are being recruited in migraine when processing sensory stimuli compared to control. Increased recruitment of the brain during stimulus processing could be an indicator of inefficiency and may be associated with increased metabolic demand [98]. Inefficient processing of visual stimuli has been suggested to be the possible mechanism for visual discomfort in nonclinical populations [99], and there is some electrophysiological evidence to support this suggestion [100,101,102]. As those with migraine report visual discomfort both during and between attacks [16,103,104], understanding oscillations and the recruitment of different brain areas during sensory processing could be important to unravelling the mechanisms of the disorder.

### 3.5. Summary

Differences in gamma-band oscillations in migraine are summarised in Table 2. Gamma-band oscillations in response to stimulation may be increased in migraine, which could indicate a lack of gain control. Furthermore, there seems to be the possibility of recruitment of additional brain regions in terms of increased connectivity in this band, which may relate to less efficient processing. This is highly speculative at present. In addition, previous research in nonclinical populations has shown the amount of spatial suppression (thought to relate to excitation–inhibition imbalance) is not related to peak gamma frequency or power per se, but it is the change in gamma power (suppression) that relates to surround suppression [105]. Therefore, more directed research into excitation and inhibition in migraine and the role of gamma-band oscillations would be advised using paradigms investigating gamma-band suppression in those with migraine directly [92].

## 4. Integrating Alpha- and Gamma-Band Oscillations

### 4.1. Thalamocortical Dysrhythmia

Although it is convenient to consider oscillatory activity in separate bands, this simplified view is unrepresentative of the activity of the brain-oscillations are likely to interact [108]. In particular, alpha- and gamma-band activity have been shown to relate to each other [109]. When the alpha band cycle is at a trough, this allows for stimulus-induced gamma burst to appear, and so gamma power increases [41]. When occipital tACS was administered both at the individual alpha frequency and at ±4 Hz of the individual’s alpha-band frequency (i.e., outside of the alpha frequency range), gamma-band oscillations were suppressed and visual task performance decreased [110]. Therefore, Herring et al. [110] suggest that the application of slower frequency oscillations, whether the individual’s individual alpha frequency or not, can suppress gamma-band oscillations. In addition, research by Orekhova et al. [111] showed that alpha-beta band suppression occurs irrespective of visual stimulus properties, and so alpha oscillations may index attentional mechanisms. By contrast, the amount of gamma band suppression was directly influenced by either contrast or velocity of visual stimuli, suggesting gamma oscillations are involved with processing the properties of the stimulus itself.

The interaction between low- and high-frequency band oscillations has been referred to as cross-frequency coupling [112] and has been suggested to represent an index of the efficiency of the system in the case of neurological disorders, including migraine. Specifically, the communication between the thalamus and the cortex is thought to be controlled by cross-frequency coupling. Llinás et al. [113] suggested thalamocortical dysrhythmia for a range of disorders, including tinnitus, depression, Parkinson’s disease, and, importantly, headache. One of the key ideas is that communication between the thalamus and the cortex is controlled by the network dynamics, which require the interaction between different oscillatory frequencies. In normal populations, slower oscillations (typically around the alpha-band frequency) periodically suppress the bursts of gamma activity that are thought to contain stimulus-specific information. There are feedforward and feedback loops between the thalamus and the cortex based on information from the thalamocortical and corticothalamic connections (afferents). Moreover, alpha-band power in the cortex is positively correlated with glucose metabolism in the thalamus measured using PET [114]. Thalamocortical dysrhythmia in these clinical populations is characterised by widespread coherent low-frequency oscillations towards the 5–10 Hz range, whereas control participants show oscillatory activity peaking more towards a faster activity around 10Hz. These increased lower-frequency coherent oscillations (5–10 Hz) in the clinical population are thought to result in increased gamma-band oscillations, which in turn are responsible for the positive symptoms of these disorders, for example, the hallucination of migraine aura. Llinás et al. [113] suggested that for some disorders, this process may be bottom-up, but in the case of epilepsy and migraine, this may have a top-down origin from the thalamocortical input. A reduction in the thalamocortical input may lead to a widespread low-frequency oscillation pattern that, in turn, is connected to excess inhibition. This increase in gamma-band activity is thought to lead to the “edge effect” that manifests as the hallucinations of the aura itself.

Conversely, Coppola et al. [25] proposed thalamocortical dysrhythmia as an account of reduced inhibition in migraine. According to this view, abnormal thalamocortical control will lead to widespread lower-frequency activity, which will in turn lead to increased cortical activity in the higher-frequency bands. The implication of this is that reduced thalamic control leads to a shift from the more usual alpha-band frequencies to lower, more theta-like frequencies, and therefore, there will be increased bursts of cortical gamma-band oscillations due to reduced suppression.

Coppola et al. [107] measured very high-frequency oscillations (450–750 Hz) in response to 4.4 Hz somatosensory stimulation. These very high frequencies are thought to index thalamocortical activity, as there is evidence that the early high-frequency oscillations are the result of spike activity in thalamocortical cholinergic fibres, and late high-frequency oscillations are the result of cortical GABAergic interneuron spiking activity. Early high-frequency oscillations were smaller in mixed migraine (MO and MA) compared to control groups, so the authors take this as evidence that thalamocortical activity is reduced in migraine. Similarly, Coppola et al. [115] indexed thalamocortical activity after somatosensory stimulation using high-frequency (450–750 Hz) oscillations and found this to be reduced in MO, taking this as evidence of disrupted communication between the thalamus and cortex in migraine. Moreover, the disrupted communication between the cortex and thalamus in migraine has been suggested to be due to increased slow oscillations (less than 0.25 Hz) in mixed migraine (MA and MO) compared to control groups, and there is evidence that these low-frequency oscillations related to headache frequency [116].

Tu et al. [117] provided fMRI evidence to support the suggestion of thalamocortical dysrhythmia in MO, showing that those with migraine spend less time in a strong default mode state compared to controls. The default mode state refers to activity recorded over several areas of the brain that occurs in resting situations when the participant is not engaged in a goal-directed task [118]. Of particular interest is the posterior cingulate cortex that might be involved with monitoring the environment for change, and interestingly, in the case of the visual areas, the default state is more closely approximated with the eyes-open compared to the eyes-closed condition [118]. Tu et al. [117] suggest their result is compatible with the idea of thalamocortical dysrhythmia in migraine: a slowing down of the alpha-band oscillations, therefore a longer window of excitation, leading to increased gamma-band oscillations.

Lisicki et al. [106] showed that alpha and gamma activity in response to visual stimulation depends on the phase of the migraine cycle. During an attack, there is increased gamma-band activity in MO, but on headache-free days, there is increased alpha power. Lisicki et al. [106] also calculated the power ratio between alpha- and gamma-band oscillations: those with a higher alpha/gamma power ratio also experienced fewer headache days. However, using wearable technology to measure EEG throughout the migraine cycle, Martins et al. [119] showed there to be no difference in absolute or relative resting power at either alpha or gamma bands in a mixed migraine group (MA and MO). However, relative beta power increased from the interictal to the ictal stage, and relative delta power decreased from the interictal stage compared to immediately after the migraine attack. It must be noted that there is a difference in oscillations at rest compared to in response to visual stimulation, and the quality of the EEG data may be poorer in wearable headsets compared to measurements in controlled, lab-based environments.

There is additional evidence of migraine cycle effects related to thalamocortical dysrhythmia. Coppola et al. [120] show reduced functional connectivity of the thalamus during spontaneous MO attacks, suggesting reduced inhibitory control consistent with the predictions of thalamocortical dysrhythmia. There is also some work on short latency afferent inhibition (SAI) in the motor system. SAI is an index of thalamocortical control (inhibition), and this inhibition is reduced between attacks, but increased interictally in MO [121]. Martinelli et al. [122] investigated the fMRI phase coherence of thalamic and “salience network” (also known as the pain matrix) activity at four stages in induced migraine attacks, finding there to be a reduced phase coherence of oscillations during the attack itself compared to before and after. There thus appears to be a particular breakdown of thalamocortical synchronisation (and therefore thalamic control) during the ictal phase compared to the others.

Importantly, recorded from V1 local field potential with visual stimulation consisting of a 1 Hz square-wave grating gamma decreases with contrast in wild-type mice, but in a mouse model of familial hemiplegic migraine, it has been shown that there is a gamma (70–90 Hz) band increase. Moreover, using a computational model to replicate the balance of inhibition and excitation, it was shown that this increase in gamma in the familial hemiplegic mouse model was likely to be due to enhanced thalamocortical synapses and enhanced excitatory synapses [123]. This level of detail from the computational model can enable specific predictions about what excitatory and inhibitory parameters are needed to replicate experimental findings, thus giving insights into the exact mechanisms of the disorder. This will be discussed in more detail below.

### 4.2. Summary

There is growing evidence that oscillatory activity is different in migraine, and this relates to perceptual performance and some clinical characteristics. This seems to relate to a homeostatic account of migraine, as there is a change in oscillatory activity at different stages of the migraine cycle, which further implicates oscillatory activity differences in the disorder. This may be characterised by the ratio of alpha and gamma-band activity at different phases in the migraine cycle: a higher ratio interictally, but a lower ratio during the attack itself. This suggests that the normal regulatory mechanisms of excitation and inhibition are different in migraine, as these oscillatory differences are thought to index cortical excitability and suppression of activity. However, to understand why these oscillations might be different in migraine, we need to consider at the molecular level what are the excitatory and inhibitory processes resulting in oscillatory activity in the brain. In what follows, we will scrutinise the most relevant neurotransmitters whose activity has been systematically shown to be altered in migraine as compared to controls.

## 5. Differences in Neurotransmitters

MRS techniques are becoming more sophisticated and more widely available in recent years, enabling the measurement of neurotransmitter levels in patients directly. Two neurotransmitters that have been linked to migraine are the excitatory neurotransmitter glutamate and the inhibitory neurotransmitter GABA. In this section, we will review the evidence for differences in glutamate and GABA levels in migraine and the evidence for how this might fluctuate during the migraine cycle.

### 5.1. Glutamate

One of the main excitatory neurotransmitters is glutamate, and there is evidence of a role of glutamate in migraine pathophysiology (Table 3). In familial hemiplegic migraine, the triggering of cortical spreading depression (CSD) is thought to be due to too much glutamate and potassium (K+), either through excessive release or through reduced reuptake, depending on phenotype [18]. It must be noted that this would be during the attack phase, but Prescot et al. [124] showed no differences in glutamate levels between 12 acute migraine (unspecified MA or MO) and 8 control participants in the anterior cingulate cortex and insula, which are regions associated with pain. However, it must be noted that these individuals were taking various medications. During a migraine-like attack provoked by hypoxia, there was also no difference in glutamate concentration [125]. Additionally, the CSD that is thought to be responsible for the initiation of the attack is not normally associated with the anterior cingulate cortex but rather the sensory regions [126].

There is also a role of glutamate in episodic migraine. In between attacks, several authors have shown evidence of increased glutamate, glutamine, or Glx (glutamate + glutamine) in migraine compared to control groups. There is an increased glutamate/glutamine ratio in the occipital cortex in mixed migraine (MA and MO) compared to control groups [127]. There is increased Glx concentration in the thalamus and occipital regions in MO [128], a higher glutamate/creatine ratio in a sample of 10 MA compared to controls [129]. In a larger sample of 36 MA, 27 MO, and 27 controls (all unmedicated), there is evidence of higher glutamate concentration in MO compared to controls, but not MA compared to controls [130]. Wang et al. [131] found higher Glx/water ratios in chronic but not episodic migraine in the periaqueductal gray. They also found that a reduced Glx/creatine ratio in the dentate nucleus was associated with higher migraine disability.

However, other authors have found no group differences in the concentration of Glx for episodic MO, chronic MO, and controls [132]. Additionally, [135] made an argument for the increased concentration of Glx in the posterior cingulate cortex in a large sample consisting of several neurological disorders, but when considered separately, there was no strong evidence of a group difference for the migraine group of 10MA and 23MO compared to controls. However, a literature review including meta-analysis showed that there is likely to be an increased glutamate concentration in migraine compared to control [133].

Although they found no group differences, Bridge et al. [134] found correlations between MRS-spectroscopy estimated levels of glutamate and response to visual stimulation in an MA group. However, Zielman et al. [130] found no relationship between higher glutamate concentration and measures of visual sensitivity, including the Visual Sensitivity Scale [136] and the Pattern Glare Test [137]. This could be because [130] had a mixed migraine group including both MA and MO, or alternatively that the Visual Sensitivity Scale is a subjective measure based on self-report. Whilst the Pattern Glare test is an instantaneous measure, this is still possibly subject to criterion effects compared to an objectively measured response to visual stimulation. Alternatively, it could be the case that there is a need to measure glutamate levels during visual stimulation rather than afterwards, as neurotransmitter levels will fluctuate with the task. There is an argument therefore for using objective measures, for example, behavioural tests of performance and electrophysiological evidence, and also measuring glutamate levels during the task itself.

### 5.2. GABA

Research has shown GABA concentration is lower in the visual (occipital) cortex in those with migraine aura [134]. Additionally, those with more severe migraine attacks also have a lower concentration of occipital GABA shortly following the attack, and this is related to the severity of headache [138]. Of additional interest is that GABA concentration in those with MO increased in this area just before the onset of the headache in a provoked migraine attack [139]. Wang et al. [131] found lower GABA concentration in the dentate nucleus in chronic migraine. Taken together, this indicates some involvement of GABA in migraine, particularly as levels fluctuate with the migraine cycle.

Wu et al. [140] found lower GABA concentration in the anterior cingulate gyrus and medial prefrontal lobe, but no differences in Glx in MO. Negative correlations were also found between GABA levels and attack frequency. However, in other studies with mixed migraine groups, the finding of reduced GABA in the occipital cortex has not been replicated [128,141,142]. Additionally, in those with less severe migraine, and using a less powerful 3T scanner, there was no difference found in the concentration of GABA between MA and control groups [143]. Recent work by Pohl et al. [144] found no differences in baseline levels of occipital GABA in a predominantly MA group recruited from a neurology clinic. A reanalysis of the clinical data of Chan et al. [141] showed there to be a positive relationship between the number of days since the last migraine attack and GABA+ levels, but no statistically significant relationship between age at migraine onset and headache frequency. It is important to note these correlations are based on very small sample sizes, and so should be interpreted with caution. In those with chronic migraine with comorbid depression, there was evidence of reduced GABA levels [145], which is important as depression is a risk factor for chronification [22].

In a paediatric sample of migraine, there were no differences in GABA in the thalamus, sensorimotor, or visual cortex [146], which does not support the idea that differences in GABA concentration are the cause of the disorder. However, they did find that glutamate concentration was reduced in the visual cortex and elevated in the thalamus in MA. They also found that higher GABA concentration was associated with worse migraine, and higher GABA/Glx ratios in those who had had migraines for longer. These results do not support the idea that differences in GABA and glutamate concentrations cause migraine; if anything, they support that these differences might be the result of experiencing migraine. However, there are many differences between the developing and the adult brain, so these comparisons should be made with caution.

In adults, Zhang et al. [147] measured GABA and Glx levels in the thalamus in chronic and episodic migraine (MO). They found lower GABA concentration in chronic migraine, but lower Glx concentration in episodic migraine compared to control groups. Additionally, they found that the GABA/Glx ratio was lower in the chronic than in the episodic group. They argued that reduced thalamic GABA contributes to the chronification of migraine. The overuse of acute medication, specifically barbiturates, is related to the progression of migraine from episodic to chronic [22]. Barbiturates are used as antiepileptic medication and enhance GABA by acting on the GABA_A_ receptor in the neocortex [148]. However, in contrast to adult populations, adolescents with chronic migraine do not typically overuse medication [149], and so this risk factor is less likely in this population. Therefore, it is possible that the changes in GABA as a result of the mediation disrupt the natural homeostatic mechanisms to prevent migraine, leading to headache.

Taken together, the works of Bell et al. [146] and Zhang et al. [147] suggest that there is an association between GABA and the progression of the disorder. In the paediatric sample, there was an association between a higher GABA/Glx ratio and the duration of the disorder, whereas in the adult sample, Zhang et al. [147] suggest that lower GABA concentration is indicative of the chronification of migraine. It is important to note these are associations and causality is difficult to establish here, and in addition, the developing brain is very different to the adult brain, so more information is needed before firm conclusions can be drawn.

Other authors have shown elevated levels of GABA+ in those with migraine (unspecified whether MA or MO) [150] in the parietal and somatosensory cortex. An increase in GABA+ concentration has also been found in the posterior cingulate gyrus in migraine, as well as other pain conditions such as lower back pain. This suggests that in this region, the increased GABA levels might be to do with the experience of pain rather than the origin of migraine [32]. It is also important to note that these authors refer to GABA+ rather than GABA to reflect that their measure could be contaminated with other metabolites.

Importantly, although Pohl et al. [144] found no differences in baseline occipital levels of GABA, they did find a reduction in GABA concentration after daily anodal tDCS of the occipital regions in a mixed migraine group (MA and MO). It must be noted that this finding was not there at the 4-month follow-up, suggesting effects are short-lived. Taken together, this suggests that excitatory stimulation in the occipital regions is related to a reduction in GABA levels, which could arguably explain the lack of inhibition, but only in relation to stimulation.

Several authors have highlighted the difficulty of interpreting causality in the relationship between GABA and migraine. One possibility is that altered neurochemical balance, in the form of reduced GABA concentration, may be a predisposing factor for migraine [151] and play a causal role in the sensory processing differences associated with the disease. In contrast, it has also been argued that changes in GABA may be a response to migraine attacks, such that GABA concentration is increased in an attempt to reduce or suppress attacks [139,152]. The results of studies of GABA in migraine are summarised in Table 4.

### 5.3. Serotonin

Glutamate and GABA are not the only neurotransmitters associated with migraine; serotonin has also been implicated as having a role in migraine pathogenesis. There is evidence to support this view, as medication to enhance serotonin has been used in migraine prophylaxis, although this is not always effective and can result in a worsening of migraine (for a review, see [153]). Additionally, tryptophans, which contain the precursors for serotonin, have been associated with migraine: dietary interventions to reduce tryptophans in people with migraine have been shown to aggravate the symptoms of photophobia, nausea and headache [154], and conversely, dietary supplementation to increase tryptophans have been shown to reduce the likelihood of migraine attack [155].

However, this may be a more complex story. Lower levels of serotonin have been shown in the plasma of those with MO, but not MA, in between attacks [156]. Importantly, imaging the serotonin 5-hydroxytryptamine (5-HT) receptor binding using PET to estimate 5-HT levels showed evidence of increased 5-HT levels in MO rather than the hypothesised reduced levels [157].

The role of serotonin in migraine is thought to relate to the trigeminal system, which is related to the experience of pain, and this has been supported by animal models [158]. However, there may also be a role of serotonin in increasing neural excitability, as there was evidence of an increase in the number of waves of cortical spreading depression with reduced serotonin [159].

To further complicate matters, there is an interaction between serotonin, glutamate and GABA. Administration of a serotonin precursor (5-hydroxytryptophan) reduces glutamate levels for a limited time in the hypothalamus of animal models [160]. Additionally, taking selective serotonin reuptake inhibitors (SSRIs) increases occipital GABA levels measured with MRS in depressed patients [161] as well as healthy volunteers [162]. The role of serotonin is unclear in migraine, and it could be that this is another mechanism, or it could be that there is an effect on glutamate and GABA levels in some individuals.

### 5.4. Summary

There seems to be a relationship between GABA concentration and migraine, specifically, lower GABA levels seem to be indicative of more severe attacks and those with chronic migraine. As there seems to be no evidence of GABA differences in paediatric migraine, it might be that GABA is secondary to the development of the disorder. Additionally, a higher concentration of GABA has been noted in the pain pathways, possibly suggesting that this is an attempt to inhibit the pain of the migraine. As before, many findings are mixed, possibly due to group inclusions and how frequent attacks are, and it might be simply more likely that those with chronic migraine will be experiencing the time period around the attack rather than the truly interictal stage. Despite the mixed evidence, there seems to be some indication of GABA imbalance in migraine sensory pathways, possibly indicative of reduced inhibition, although this is subject to whether this is at rest or during visual stimulation, and possibly the point of the migraine cycle. In addition, there are other neurotransmitters that are associated with migraine, for example, there is an important role of serotonin in migraine, but it is speculative at present to comment on this.

## 6. Excitation–Inhibition Balance and Dysrhythmia

The story so far is that episodic migraine seems to have some sort of disturbances in oscillatory activity, also termed dysrhythmia. In addition, there is growing evidence that excitatory and inhibitory neurotransmitters may be different in migraine, resulting in an imbalance between excitation and inhibition. In this section, we will review how an imbalance in neurotransmitters may relate to dysrhythmia.

### 6.1. Relationship between Oscillations and Neurotransmitters

#### 6.1.1. Glutamate and Oscillations

There may be a relationship between alpha-band oscillations and glutamate levels [163]. Using computational modelling, it has been shown that alpha oscillations in thalamocortical neurons depend on acetylcholine, whilst thalamoreticular neurons depend on glutamate [164]. Additionally, there is pharmacological evidence of a role of glutamate in alpha-band oscillations. At high levels, ketamine is thought to have an anaesthetic effect by blockading the N-methyl-D-aspartate (NDMA) receptors for glutamate. However, at low levels, ketamine increases glutamate transmission by acting on non-NDMA receptors [165], and low levels of ketamine result in a decrease in alpha power [166,167].

#### 6.1.2. GABA and Alpha-Band Oscillations

Moreover, increasing GABA function decreases occipital alpha-band power both at rest and during visual tasks (for a review, see [168]). Benzodiazepines are medications that enhance GABA by binding to the GABA_A_ receptor, and there is evidence that taking benzodiazepines suppresses resting-state alpha-band oscillations [114]. Enhancing GABA using benzodiazepines results in a reduction in the stimulus modulation of alpha-band oscillations and also poorer performance on a taxing visual working memory task [169]. This is important as these modulations of alpha-band oscillations during a task are thought to be integral to task performance. If those with migraine do not modulate alpha-band oscillations in the same way as controls, this might manifest as poorer performance on similar tasks.

There is evidence that alpha-band oscillations have been associated with GABA; however, much of the literature is indirect, for example, using medications to influence GABA. Medications may have several effects, as well as influencing GABA, and therefore results should be interpreted with caution. There are few studies investigating direct measurements of GABA concentration and the relationship with alpha parameters such as peak frequency and power, even in nonmigraine populations. One such study conducted by Baumgarten et al. [170] showed a positive relationship between MRS-estimated GABA concentration and peak alpha frequency, where those with higher GABA concentrations were also those with higher peak alpha frequency. This relationship was only seen when the patient group with hepatic encephalography were combined with the control group. It is possible this additional power was needed due to the small sample in MRS studies, especially with liver-related brain disorders. If GABA levels are lower in migraine, it might be expected that the peak alpha frequency is slower. A study combining MRS and EEG would be needed to demonstrate this, taking into account the migraine cycle and also measurements at rest as well as during a visual task. This would bring together existing knowledge into testable hypotheses.

#### 6.1.3. GABA and Gamma-Band Oscillations

Gamma oscillations are thought to be controlled by alpha-band oscillations, which act as an envelope of suppression, but what are the generators of gamma-band oscillations? Gamma oscillations themselves are thought to be generated through excitatory and inhibitory processes, specifically including GABA [171]. It has been suggested that these oscillations arise from interneurons connected by GABA_A_ synapses that have a tonic excitation, the decay constant of the inhibitory postsynaptic potential determined the frequency of the oscillation [172]. There is evidence of reduced gamma oscillations with asynchronous release of GABA [173]. Additionally, Muthukumaraswamy et al. [174] found that the frequency of gamma oscillations increases, and the fMRI response decreases with increased GABA concentration (the gamma band was defined as 30–100 Hz, but with peaks ranging between 40 and 66 Hz). This positive relationship between gamma oscillation (60–90 Hz) peak frequency and MRS-estimated GABA concentration has also been shown in the motor cortex [175]. Importantly, Muthukumaraswamy et al. [174] also reported that lower frequency of gamma oscillations involved a larger area of the cortical network to be involved in the response, and greater spread of excitation through the cortex, which could be a mechanism for the widespread cortical activation thought to relate to migraine attacks.

However, there have been issues with replicating the original findings of Muthukumaraswamy et al. [174] as a large-scale study of 50 individuals failed to replicate the relationship between occipital gamma oscillations (40–80 Hz) and MRS-measured GABA concentration [176]. However, it should be noted that in the replication of Cousijn et al. [176], there were 90 stimuli presented compared to 200 in the original Muthukumaraswamy et al. [174] study, and as gamma oscillations are quite small, the limited repetitions may go some way to explain the lack of replication here. Similarly, Wyss et al. [177] also failed to find a relationship between peak gamma frequency and MRS-estimated GABA concentration in the primary auditory cortex. It could be that the effects are specific for V1 or that this study also had 78 trials, so again it may have been an issue with fewer trials and so poorer signal-to-noise ratio compared to Muthukumaraswamy et al. [174]. It has also been suggested that there are difficulties due to contamination of MRS-estimated GABA levels; therefore, Kujala et al. [178] used PET to measure the GABA_A_ receptor density in V1 and found this to correlate positively with peak frequency and negatively with the amplitude of gamma-band oscillations (40–100 Hz, peaking at 65 Hz).

#### 6.1.4. GABAergic Medications

If those with migraine have reduced GABA levels, it might be thought that medications that enhance GABA will have a beneficial effect. GABAergic medications such as muscimol and baclofen have been shown to reduce neuronal activity induced by glutamate in animal models [179]. In a Cochrane review, sodium valproate, which increases GABA by preventing its reuptake, has been shown to have beneficial effects in reducing the frequency of migraine attacks compared to placebo [180]. Benzodiazepines are thought to increase GABA through binding to the GABA_A_ receptor, and importantly the benzodiazepine lorazepam has been shown to reduce acute migraine symptoms, specifically to reduce photophobia when used in conjunction with ibuprofen [181]. However, there is some evidence that benzodiazepines increase the risk of migraine rather than reducing it [182]. Additionally, benzodiazepines, which are thought to enhance GABA, also increase gamma power [169]. Care must be taken when using medications as experimental evidence as there are several uncontrolled factors in observational studies, and also there are several other modes of action for these medications, as they tend not to be specific.

There is also a role of glutamate in gamma-band oscillations, as low levels of ketamine, thought to enhance glutamate by acting on non-NDMA receptors [165], results in increased gamma-band power [167,183]. Behavioural studies have shown a positive relationship between evoked gamma-band oscillations (30–70 Hz) and glutamate concentration in the lateral occipital areas during a task to categorise line drawings of objects or abstract images [184]. It is important to note that pharmacological interventions to increase glutamate can be counteracted by increasing levels of GABA, and so it may be the case that the reverse is also true. Therefore, it is not trivial to determine whether the origins are from reduced GABA, increased glutamate, or a simultaneous combination of the two.

### 6.2. Summary

Taken together, there is evidence of an imbalance between excitation and inhibition in migraine. One possible mechanism is that GABA may be reduced, and if so, slower alpha-band oscillations and increased gamma-band oscillations would be expected. This is what has been suggested by the theory of thalamocortical dysrhythmia. However, if GABA is lower, it could be argued that higher alpha power would be expected, as GABA agonist zolpidem has been shown to reduce alpha power [185]. Therefore there would be more conservative responses to both signal and noise, contrary to the predictions of hyperexcitation [27]. Another issue with making direct predictions is that GABA and glutamate levels are interrelated, and so manipulating one may have effects on the other; for example, glutamic acid is involved in the synthesis of GABA [186].

It is not easy to make predictions about complex systems, as there can be counterintuitive effects of interventions on oscillatory behaviour, and so it has been suggested that appropriate modelling of the system can be of benefit [187]. It is possible to model oscillatory behaviour from excitation and inhibition. This has previously been applied to migraine in work based on the cortical spreading depolarisation, activity thought to relate to the onset of hallucinations during migraine aura (for a review, see [9]). However, as well as modelling the aura mechanism, it may be possible to model the excitatory-inhibitory imbalances in migraine in more general terms, not specifically the aura stage. This will then have the benefit of being able to make precise predictions about oscillatory activity and possibly even the origin of the attack itself.

## 7. Models and Behaviour

Despite the abundance of research on the pathophysiology of migraine, there is still a scarce understanding of the relationship between molecular and cellular mechanisms, electrophysiological responses, and sensory processing. In other words, within the broader framework of the excitation–inhibition imbalance, it is not clear how alterations in neurotransmitter concentrations and changes in oscillatory patterns impact sensory processing as measured psychophysically and behaviourally. Given the complexity of these interactions, the application of mathematical models may play a crucial role in providing an integrative theoretical framework, allowing us to develop testable hypotheses about the effects of neurochemical imbalances and oscillatory modulations on sensory processing. These excitation–inhibition models can be used to make concrete predictions about task performance [188] and electrophysiological responses [189]. In this section, we review current models of the generation of oscillatory activity in the occipital cortex and what the functional significance of these oscillations is with respect to behaviour. We will also review the effects of changes in GABA/Glx on these modelled oscillations and how these models have been applied to our understanding of migraine.

### 7.1. Functional and Physiological Models

Both functional and physiological models of EEG oscillations have been developed [168]. In practice, EEG measures the postsynaptic potentials of pyramidal neurons, which need to be synchronously activated to create the oscillations that form the EEG signal [190]. In the case of alpha-band oscillations, functional models help to explain their role in inhibiting sensory information. Physiological models account for how rhythmic activity arises in pyramidal neurons and synchronises across neurons. Furthermore, physiological models enable qualitative predictions about the effects of neurochemical imbalances on the dynamics of oscillatory activity.

Models may be developed at different scales of analysis. The microscopic level models the activity of individual neurons or small groups of neurons. The mesoscopic level models the behaviour of large groups of neurons. Finally, the macroscopic level models at the scale of the whole brain, taking account of connections via the white matter [190].

**Figure 2:**
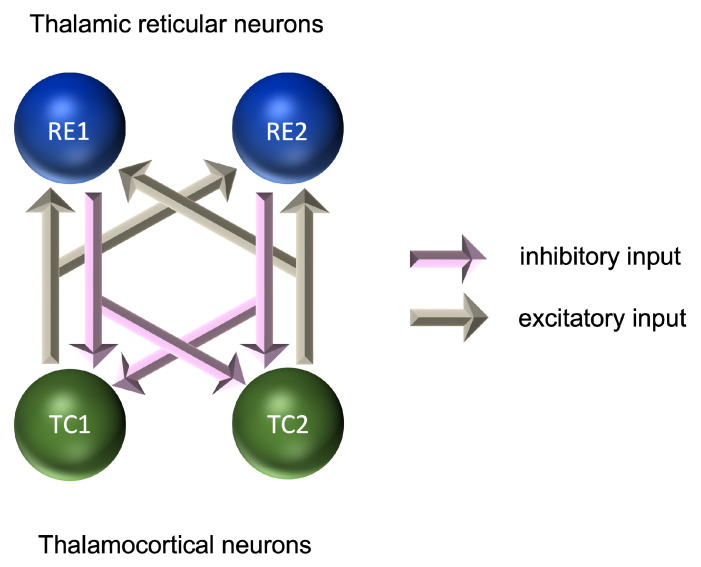
Microscopic model of interactions between neurons in the generation of oscillatory activity.

At the microscopic level, models of rhythmic activity in thalamocortical (TC) and thalamic reticular (RE) cells have been developed based on Hodgkin–Huxley models [191] of the initiation and propagation of action potentials [192,193]. RE cells provide a layer around the outside of the thalamus and are a source of GABAergic inhibition to the TC cells. Microscopic level models account for the effects of calcium and sodium/potassium dependent currents and can explain the emergence of rhythmic activity in the delta range (0.5–4 Hz) for TC cells and in the alpha range (7–12 Hz) for RE cells. These models were further developed into pairs of TC-RE cells, in which glutamatergic excitation of RE cells by TC cells is coupled with GABAergic inhibition of TC cells by RE cells (Figure 2). These pairs provide a model of the thalamic source of alpha-frequency activity, although both thalamic and cortical sources are believed to play an important role in the generation of the alpha frequency [164,168,194]. These models require inhibitory and excitatory connections; however, the relative contribution of each to the formation of the EEG signal is still the subject of investigation [190,195].

Coupled excitation–inhibition models can also be used to account for the activity of larger populations of models at the mesoscopic level. Neural mass models, based on the Wilson–Cowan equations [196], predict activity as a function of time. This is extended to account for spatial effects in neural field models [190]. These models consist of pairs of excitatory and inhibitory populations of neurons, with synaptic connections both within and between each population. As with microscopic level models, these allow us to model the influences of glutamatergic excitation and GABAergic inhibition on the dynamics of neural activity [197].

While the macroscopic level is beyond our scope, function on the scale of the whole brain may also be different in migraine. For example, there are several studies showing increased functional connectivity in migraine in both children [198,199] and adults [200]. Additionally, it should be noted that an increase in both the strength and density of connectivity has been found in migraine, including in areas associated with visual processing and the perception of pain [201].

### 7.2. Modelling GABA and Glutamate

The role of GABA and glutamate in these models means that it is possible to model the effects of differences in the levels of these neurotransmitters. For example, the fact that alpha oscillations reflect functional inhibition has led researchers to predict that increasing the level of GABA, an inhibitory neurotransmitter, should increase alpha power. In fact, the opposite is found, and pharmacological enhancement of GABA efficacy tends to decrease alpha power, both at rest [202] and during a visuospatial working memory task [169]. This discrepancy may reflect the importance of distinguishing between functional and physiological inhibition [168]. While alpha oscillations are thought to reflect functional inhibition, they are generated as a result of both excitatory and inhibitory neural interactions [163,203,204,205,206]. It is thus the balance between excitatory and inhibitory activity that must be considered when making predictions about the effects of neurotransmitter levels on oscillatory activity [169,207].

This highlights both the centrality and the complexity of computational modelling in this field [168]. While models allow us to make quantitative predictions about the dynamical activity of neural populations, these predictions require the levels of all the relevant parameters to be considered. This means that broad qualitative predictions, such as the effects of altering GABA efficacy on the amplitude of alpha oscillations, may not correspond with our intuitions.

This can be seen when these models are applied to migraine. On the one hand, the relationship between predicted differences in GABA concentration and alpha power is in agreement with other results. That is, evidence points to reduced GABA levels [134,138,140,147], and increased alpha power [49,51,52] in migraine. This is consistent with the effects of direct manipulation of GABA efficacy [169,202]. On the other hand, psychophysical tests of the effects of altered excitatory–inhibitory balance in migraine, in the cases of binocular rivalry and surround suppression, have tended not to produce results consistent with predictions.

### 7.3. Models of Binocular Rivalry

**Figure 3:**
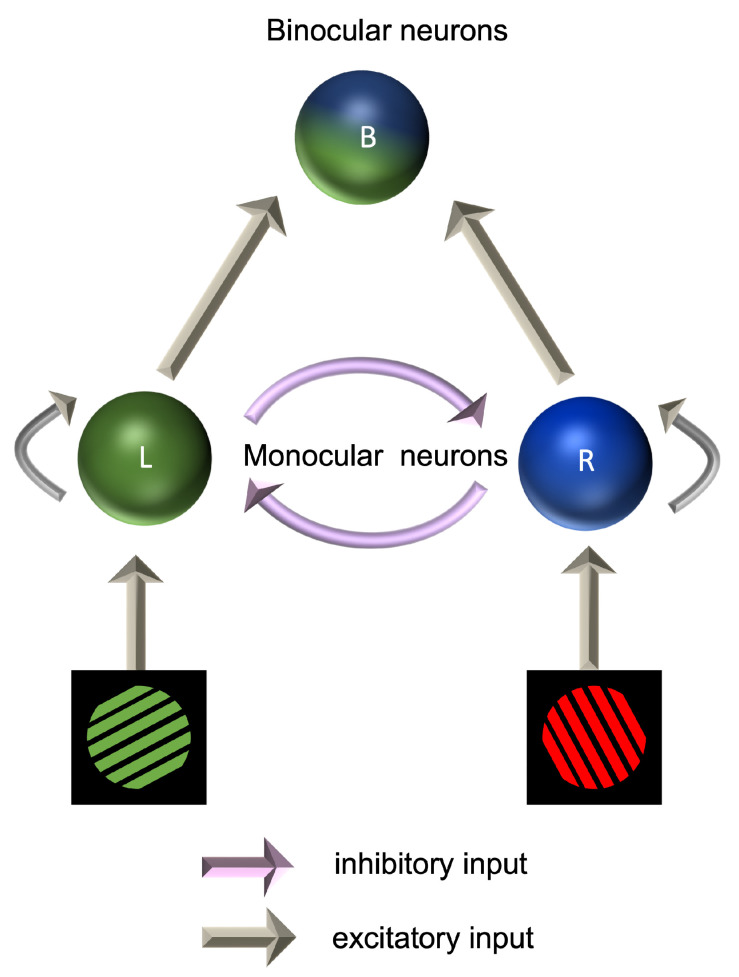
Mesoscopic model of interactions between populations of neurons in binocular rivalry.

Binocular rivalry occurs when two dissimilar images are presented to the two eyes. Rather than fusing the two images into a composite percept, observers tend to report seeing one or the other, with this flipping between the two over time [208]. As the brain alternates between monocular suppression and dominance, there is competition between the two eyes for perceptual dominance [209]. Monocular neurons are thus receiving excitatory inputs from one eye and also inhibitory inputs from the other eye. Dominance is dictated by whichever eye generates the strongest inhibition towards the other eye. The dynamics of binocular rivalry have been modelled as a Wilson–Cowan excitation–inhibition system (Figure 3). In this model, the responses of monocular populations of cells are amplified by recurrent excitatory connections, while the suppression of the input from the unseen eye is mediated by interocular suppression [210]. Under this model, an increase in either the strength of recurrent excitation or interocular suppression would lead to longer periods, during which one eye’s image was perceived before switching to the other eye. Reduced inhibition in migraine would therefore be predicted to lead to more rapid switching times. This predicted difference in the dynamics of binocular rivalry has not been found [142]; if anything, switching occurs less rather than more frequently in migraine [211,212].

The difficulty in making predictions at the perceptual level that arise from neurochemical differences can, however, be seen when we consider models of physiological and functional inhibition. Individual differences in GABA concentration have been shown to be related to the dynamics of binocular rivalry. Individuals with greater occipital GABA tend to show greater levels of suppression [213] and shorter durations of the dominant percept [214]. Moreover, pharmacological enhancement of GABA efficacy produces similar results [215]. However, binocular rivalry is not related to alpha power [216], contrary to predictions from the fact that increased GABA levels are related to reduced alpha power [169,202]. Binocular rivalry is, however, related to alpha frequency, with higher frequencies tending to be related to more rapid switching rates [216]. The reduced rate of switching in migraine might be expected to be related to a lower alpha frequency. Neufeld et al. [61] found the opposite, a faster peak alpha frequency in migraine compared to control groups in between attacks, although alpha frequency does tend to reduce over time in migraine, such that those who have experienced migraine for longer have a slower alpha-band peak frequency [47,62]. Disease duration thus also needs to be taken into account when comparing performance on psychophysical tasks.

### 7.4. Models of Surround Suppression

Surround suppression has been used as a spatial measure of suppression. When presented against a high-contrast surround, the apparent contrast of a target tends to be reduced. If spatial inhibition were reduced in migraine, then lower levels of surround suppression would be expected. Surround suppression tends, however, to be greater in migraine [217]. While this result was the opposite of that predicted by the authors, they concluded that models of surround suppression need to take account of both excitatory and inhibitory interactions. This again highlights the need for fully developed models of excitatory-inhibitory interactions in sensory processing in order to predict perceptual differences in migraine. The results of a recent dietary intervention study [218] further complicate this conclusion. In this study, high doses of vitamin B6, which were expected to increase GABAergic neural influences, possibly through increased GABA synthesis [219], led to an increase in surround suppression. The increased surround suppression in migraine is therefore not consistent with a simple model of reduced GABAergic activity.

### 7.5. Modelling Other Visual Processing Differences in Migraine

Wilson–Cowan models have been shown to predict a variety of contrast and orientation effects in normal vision [220]. This is important because in migraine there have been demonstrable differences in contrast sensitivity (e.g., refs. [104,221,222], as well as orientation discrimination [223] and motion perception, e.g., ref. [224]. By using these models, it may be possible to replicate the migraine brain by changing specific parameters related to excitation and/or inhibition.

### 7.6. Modelling the Effects of Neurostimulation

It has been suggested that complex oscillatory systems might also be modelled using coupled oscillator models to predict the effects of neurostimulation on the brain’s ongoing activity [187]. Weak amplitude tACS can result in either increased or decreased oscillatory entrainment, depending on the strength of baseline synchronisation-weak tACS applied to highly synchronised activity results in decreased entrainment rather than increased entrainment. However, if the amplitude of tACS is increased to overwhelm the baseline levels, this can then increase entrainment, even in highly synchronised oscillations. Importantly, these complex effects were successfully replicated using a simple oscillatory model, called a Stuart Landau model [225]. Therefore, as well as during the migraine aura, it may be possible to investigate the effects of neurostimulation on migraine using mathematical models, to be able to make predictions about whether this is likely to work as a therapy.

### 7.7. Summary

In summary, excitatory-inhibitory models have the potential to link putative differences in glutamatergic excitation and GABAergic inhibition to perceptual differences in migraine. While the predictions of these models have not always been borne out, for example, in studies of binocular rivalry and surround suppression, there is good reason to believe that this might in some cases reflect the incorrect formulation of the predictions of these models rather than an absence of the expected physiological differences in migraine. Given the complex dynamical nature of the models, detailed computational modelling is necessary to fully understand the effects of neurochemical imbalances on their behaviour. These models are likely therefore to form an increasingly important component of our understanding of sensory processing in migraine in future research.

## 8. Treatments

Treatments have mainly focused on symptomatology, and mostly attempting to alleviate pain. Clearly, a better understanding of the pathophysiology of migraine may significantly improve the way to effectively intervene in migraine. Several attempts have been made to understand the mechanisms of action both at the molecular level and at the system level to propose either pharmacological or neurostimulation treatments. However, there is little understanding of how to tailor treatments to the individual needs. The understanding of the multifactorial dimensions explaining the genesis of a migraine attack becomes a very relevant challenge for translational and clinical research in this field. In this section, we will review the current knowledge and perspective on pharmacological and neurostimulation treatment currently available and their impact and limitations.

### 8.1. Pharmacological Interventions

As we have learned from previous sections (see Section 5), there is evidence that there is a neurotransmitter imbalance in migraine. It may seem logical to redress this balance with medication, although current medication does not always work for all, and patients have reported being dissatisfied with treatment [226]. There are also side effects, especially in the case of antiepileptic medication and beta-blockers [227]. In terms of specificity, there have been recent advances with medications that inhibit calcitonin gene-related peptides (CGRPs), and there is evidence from clinical trials that they are well-tolerated [228]. CGRPs are neuropeptides that are released around the trigeminal ganglion [229], which is associated with the pain in migraine, and the theory is that medication blocking these will help to alleviate the pain [230]. There are several types, including gepants, triptans, and the much larger molecules of monoclonal antibodies [231]. Medication blocking CGRP has been shown to be more effective than other preventative medications for migraine; however, there is limited evidence of long-term issues [157].

### 8.2. Nutritional Interventions

It may be possible to modify neurotransmitter levels, and therefore the balance of excitation and inhibition, through dietary interventions. Vitamin B6 is thought to be a precursor for GABA synthesis in the brain [219,232]. In nonclinical populations, those who consumed a substance relatively rich in vitamin B6 (Marmite) for a month showed reduced electrophysiological responses measured using EEG compared to the control (peanut butter) [233]. Marmite contains a selection of vitamins, but it has also been demonstrated independently using vitamin supplements that vitamin B6 reduces contrast surround suppression in nonclinical populations [218]. Additionally, vitamin B12 showed a nonsignificant trend in reducing contrast surround suppression [218], and vitamin B12 is associated with GABA increase and glutamate decrease in animal models [234].

There is evidence that dietary vitamin supplements help with migraine. A meta-analysis of the research showed vitamin B2 (riboflavin) to have beneficial effects in reducing the frequency of attacks when taking 400mg a day for 3 months [235]. It has been suggested that vitamin B2 is beneficial in migraine by counteracting mitochondrial dysfunction leading to abnormal oxidative metabolism in migraine [236]. However, vitamin B2 is a precursor to vitamin B6, which helps to convert glutamate into GABA [237], thereby increasing inhibition and decreasing excitation at the same time.

Moreover, injection of vitamin B6 prevented seizures in a case study of a young child with low levels of GABA [238]. This is important in the case of migraine as there have been parallels drawn between seizures and migraine attacks [239].

Therefore, B vitamins (and possibly others) may be of interest as a relatively low-risk dietary intervention suitable for many who may be contraindicated for other interventions to redress the excitation–inhibition balance in migraine. Large-scale, placebo-controlled studies with both experimental (performance) and clinical outcomes (migraine diary of headache frequencies) are needed. Importantly, if vitamin B6 affects the levels of GABA and glutamate, then this should have an effect on the ongoing oscillations, specifically alpha and gamma-band oscillations. There is evidence that insufficient vitamin B6 leads to abnormal EEG patterns [240] and seizures [241]. However, as yet, there is little research on the effects of taking vitamin supplements on oscillatory activity in migraine specifically, and so this is an important future direction, as well as considering the clinical outcomes.

Treatments involving vitamin supplementation might be one way of redressing the balance between excitation and inhibition in the brain in terms of directly changing the neurotransmitter levels. Treatments involving neurostimulation may also be effective in affecting the balance of inhibition and excitation by changing the dysrhythmia thought to be the origin of migraine. Specifically, tACS can be used to increase the speed of alpha-band oscillations, and this has been shown to impact behaviour [60]. If the alpha band is characteristically slower in migraine, it could be the case that increasing the speed using neurostimulation may be of benefit. Similarly, it may be possible to influence gamma-band oscillations using neurostimulation, potentially tRNS. As the gamma-band oscillations are broader band compared to alpha-band oscillations, with possibly two peaks [89] then broader band stimulation is needed. Importantly, there is some evidence that hf-tRNS can modulate gamma-band oscillations [242,243].

### 8.3. Neurostimulation Interventions

Single-pulse TMS (sTMS) has been approved for use in acute migraine attacks by the FDA. The treatment is to apply either single or double pulses up to a maximum of 16 in total per day to the occipital cortex and has beem shown to have benefits reducing pain and other symptoms of migraine such as nausea [244]. In addition to sTMS, transcranial electrical stimulation (tES) could be a valuable technique for shaping the underlying mechanisms of migraine. tES methods are far less powerful and are thought to act on changing the membrane potential, thus increasing/decreasing the probability of a response rather than directly invoking them. Therefore, tES may be more suitable as a prophylactic treatment, particularly as several sessions are typically needed to see lasting effects (for a review, see [245]). However, the exact mode of operation is currently unclear. For direct current stimulation (tDCS), it is thought that anodal stimulation has excitatory effects, and cathodal stimulation has inhibitory effects. If the issues with migraine are related to increased excitation, then cathodal stimulation of the visual cortex may help to reduce the likelihood of attacks. There has been evidence that this is the case, including randomised controlled trials [246,247]. Other authors have argued that increasing the excitation in the areas that are involved with the control of pain would be beneficial, and these are the more anodal areas of the brain. Evidence has also been shown in support of this from randomised controlled trials [248,249], but there is evidence that cathodal stimulation also is beneficial [250,251].

However, as previously mentioned, the effects of neurostimulation may depend on the level of synchronisation of the ongoing oscillations, the amplitude of the stimulation [225], and the frequency of the stimulation relative to the ongoing oscillations [60]. Therefore, modelling the effects of neurostimulation on migraine prior to experimentation would be of benefit, as it may be expected that the effects of neurostimulation may well be different compared to control groups. If migraine is primarily related to dysrhythmia, then it may be the case that alternating current stimulation (tACS) could be of benefit. tACS can be used to modulate alpha-band power (for a review see [252]). Importantly, tACS can be used to modify alpha-band oscillations and thus influence performance on a perceptual task [60]. Using tACS to test the theory of thalamocortical dysrhythmia would potentially be possible, although as the thalamus is a relatively deep structure, then care would need to be taken to stimulate this area only. To date, there are very few studies using tACS in migraine. Antal et al. [253] used 140 Hz tACS over motor areas as an acute treatment for migraine and showed a reduction in pain severity compared to sham. However, more targeted studies might use other frequencies, based on computer simulation, to drive oscillations and see if the dysrhythmia can be redressed using neurostimulation. As well as stimulating the cortex using a particular frequency of tACS, there is also the possibility of using broader band noise stimulation such as transcranial random noise stimulation. This is thought to work through stochastic resonance processes, and the small amount of stimulation close to the perceptual threshold is sufficient to boost the signal and therefore increase performance [254,255]. There is very little literature on the effects of tRNS on migraine. One study showed there to be an improvement for both a mixed migraine (MO and MA) and control group with the hf-tRNS stimulation compared to sham stimulation [256]. This was an experimental study and did not report clinical characteristics; however, given an effect on the visual perception task, this seems to imply that this may also be a stimulation type worth investigating.

Additionally, neurostimulation may also affect neurotransmitter levels, for example, anodal tDCS reduced GABA+ concentration in the dorsolateral prefrontal cortex [257] and motor cortex [258]. Therefore, this could represent a second mode of action for neurostimulation in migraine, either by changing the oscillations or by changing the neurotransmitter levels, or possibly both. Whilst this may pose an experimental problem in terms of confounding variables, if it is possible to use nonpharmocological means to change neurotransmitter levels, then this may be more acceptable to patients who are otherwise unable to take medications.

### 8.4. Summary

In summary, treatments currently employed to treat migraine mainly target the manifest symptom (i.e., pain). New interventions based on pharmacology, dietary supplements, and neurostimulation may make a step further as they point toward modifying the underlying causes of migraine. Reviewing the state of the art, it seems apparent that neurostimulation and vitamin supplementation have the potential to influence the excitation–inhibition balance in the brain and therefore possibly provide effective prophylactic therapy that is available to those who cannot take medication or for whom the medication is not effective. However, at present, this is speculative and more large-scale, tightly controlled studies on clinical outcomes of migraine are needed.

## 9. Discussion

Migraine is one of the most frequent and debilitating neurological disorders in the general population. In this review, we have shown that numerous lines of research suggest that migraine symptoms derive from a dysregulated trade-off between cortical inhibition and excitation. This dysfunctional mechanism would, in turn, result from complex interactions between neurotransmitters and oscillatory disruption. To sum up, multilevel alterations have been shown in migraine, extending from the neurotransmitter profile to manifest behaviour in the form of symptoms. In particular, alterations in GABA and glutamate concentrations have been found, as well as disturbances in alpha and gamma oscillations that could, in turn, stem from a dysrhythmia between thalamic and cortical activity. However, the evidence is very mixed and often conflicting. One of the reasons underlying this heterogeneity may lie in the fact that different types of migraine have different underlying triggers. For example, alpha-band phase synchronization has been found to be increased during SSVEP flash stimulation in MO but not in MA patients [79] and the presence of higher glutamate levels characterised only patients with MO, but not with MA [130]. Similarly, the phase of the migraine cycle could be a critical factor accounting for the conflicting findings. In this respect, there is a need for more precise instruments that can accurately determine the migraine phase as it is currently not always feasible to differentiate, for example, between an interictal and a preictal phase.

### 9.1. Excitatory–Inhibitory Imbalance as a Homeostatic Mechanism

One of the points on which researchers appear to agree concerns the framing of migraine as a disorder of cortical excitability/inhibition. However, the directionality of the effect is not still clear. For example, there are some indications of a GABA concentration imbalance in migraine [147], indicative of reduced inhibition, but contrary evidence has also been reported such as an increase in alpha power during the attack phase that would testify to reduced cortical excitability [46,47]. An important point to emphasise is that overinhibition and overexcitation may characterise migraine in a different fashion depending on the phase considered. For example, during an aura attack, there might be cortical overexcitation, especially in visual regions, while between phases, there might be a rebound of overinhibition. These cyclic imbalances between excitation–inhibition might be viewed as a dysfunctional attempt to maintain a homeostatic balance, switching from periods of hyper-excitability to refractory periods of hypo-excitability. Moreover, this altered homeostatic process may change as the migraine advances: a rebalancing could lead toward a resolution of migraine attacks, while an inability to dynamically maintain homeostasis could lead to chronicization of the disorder. Furthermore, it should be noted that, in a system so integrated as the human brain, processes that occur locally have a repercussion in other cortical regions that are connected to them. This implies that inhibition in one cortical area might cause overexcitation in a functionally related area. For this reason, it is also possible that overexcitation and overinhibition coexist simultaneously during the migraine phases in a different fashion considering the specific brain sub-region.

### 9.2. Excitatory–Inhibitory Imbalance and Sensory Processing

Another aspect currently deficient in the migraine literature, for which further studies will be needed, involves the investigation of the relationship between sensory processing and the imbalances in brain neurophysiological activity. This aspect assumes importance because there are several peculiarities in perceptual processing in migraine patients. For example, photophobia (aversion to light) during an attack is one of the diagnostic criteria for migraine, and visual stimuli, such as bright lights and striped patterns, could be the primary trigger for an attack. These symptoms can be linked to theoretical lines which conceive the sensory system of migraine patients as hyper-reactive [25] and hyper-excitable [27]. The overexcitation of the visual cortices could result from an abnormal synchronisation in the alpha oscillations as they are responsible for regulating brain excitability [41]. In addition, recent lines of research have demonstrated that the amplitude of alpha oscillations is related to visual awareness [259] and strong prestimulus alpha-band power resulted in a suppression of early ERP components [260]. Therefore, an excessive alpha desynchronisation could lead to an increased amplification of sensory signals that, in turn, could cause the visual disorders subjectively reported by patients. A recent work reinforces this interpretation [69], as it showed that migraine patients have both significantly lower alpha power before stimulus presentation than controls, and significantly higher alpha desynchronisation after the stimulus onset.

In addition, converging evidence showed that migraineurs exhibit gamma band hyperactivity in response to sensory stimulation [28]. Following the predictive coding framework, gamma oscillations appear to encode stimulus-based information and prediction errors [261].

The combination of reduced alpha oscillation amplitude and increased synchrony in the gamma band might account for the hyper-responsiveness of the visual cortex and sensory overprocessing that may foster the onset of visual symptoms such as visual aura and photophobia. It is worth noting that the same pattern consisting of decreased alpha amplitude and increased signalling in the gamma band has also been observed in the autism spectrum (ASD) [262,263,264,265,266], in which they have been conceived as a possible cause of the excessive sensitivity to environmental stimuli typically observed in the ASD spectrum [38]. Interestingly, children with autism spectrum disorders who experienced migraine showed greater sensory hyper-responsiveness than those without migraine [267], suggesting that altered sensory processing may act as a facilitator of migraine onset.

However, as shown in the review, there are numerous opposing results in which synchronisation in alpha appears to be increased [49,51], which points to the presence of an opposite phenomenon related to increased cortical inhibition. Nevertheless, when considering the homeostatic maintenance deficit hypothesis, these contradictions are only on the surface: the “hyper-reactivity” and “hyper-excitability” of the perceptual system (i.e., when the alpha amplitude is low) might characterise only certain periods of the migraine cycle, while there might be an “overinhibition of return” (i.e., when the alpha amplitude is high) afterwards. Additional work is needed to clarify which may be the internal/external triggers that can turn the system from one state to another.

### 9.3. Imprecise Perception in Migraine

In addition to being overreactive, the evidence presented indicated that the perceptual system of migraine patients is also more imprecise, which could result from noisier sensory encoding. For example, studies have found altered contrast sensitivity in migraine sufferers compared to a control group [104,221], which has been suggested to be due to increased neural noise [268]. Additionally, participants with migraine exhibit higher motion-coherence thresholds than controls [224] and increased internal noise estimates on motion perception tasks [256]. Moreover, in a binocular rivalry task, the rate of switching between the two images is lower in migraine patients [211,212]. These low-level perceptual deficits could result from the slowing down of alpha oscillations often found in migraine patients [47]. Indeed, Individual Alpha Frequency has been implicated in the precise encoding of perceptual evidence [58,59,269,270], in the segregation of sensory information [60,271,272,273], and slower alpha speed has been connected to less rapid switching rates between the two images in a rivalry binocular task [216]. Thus, a slower alpha pace would lead to a poorer encoding of external information resulting in decreased perceptual abilities. However, future studies are required to clarify whether deficits in basic perceptual processing in migraine patients may be mediated by the slowing of alpha oscillations.

### 9.4. Potential Role of Rhythmic Neurostimulation Protocol to Restore the Altered Oscillatory Process

A better understanding of the pathophysiology of migraine may significantly improve the way to effectively intervene in migraine and may foster a paradigm shift from the current one that focuses on the elimination of symptomatology, mainly through painkillers that can relieve suffering. Thus, understanding the multifactorial dimensions that account for the genesis of a migraine attack becomes a very important challenge for translational and clinical research. For example, the evidence for the existence of altered cortical oscillations as a possible driver of migraine onset calls into question the full range of techniques, neurostimulation in the first place, that can intervene and rebalance altered markers.

Taken together, there seems to be evidence that there is a case for dysrhythmic neural oscillations and neurotransmitter imbalances as a physiological marker of migraine. In future work, it would be worth investigating whether pharmacological interventions reflect a change in these physiological markers. For example, there has been much progress with medications to reduce the effects of CGRPs as a prophylactic in treating migraine. The effects of CGRP release have been thought to be secondary to disturbances in neural oscillations between the hypothalamus and other sites [230]. However, if neurotransmitter imbalances are a homeostatic mechanism, reducing the effects of CGRPs may also affect the levels of the other neurotransmitters, but it is unclear whether this is the case. Future research may be directed at testing whether symptoms of migraine are reflected in a change in these physiological markers of dysrhythmic oscillations and neurotransmitter imbalances.

## 10. Conclusions

Despite the conflicting results of experimental studies, there seems to be a picture emerging of migraine as being characterised by disordered neural oscillations, in particular, those related to thalamocortical dysrhythmia have slower alpha-band oscillations and increased gamma-band oscillations. In addition, there seems to be evidence of neurotransmitter imbalance in migraine, possibly related to homeostatic mechanisms. Although not specific and reliable enough for diagnosis, these physiological indicators point to an imbalance of inhibition and excitation in migraine, although the exact nature of this is not well understood. By using mathematical modelling, it will be possible to make testable predictions linking these inhibition and excitation imbalances to neurotransmitters and neural oscillations, as well as making firm predictions about behavioural performance on tasks of visual perception. These predictions can also be used to devise alternative treatments for those who cannot or do not wish to take medications.

## Figures and Tables

**Table 1:** A summary of the main MEG/EEG studies showing alteration in alpha oscillations in migraine.

Study	Summary of Findings	Paradigm	Sample
Hall et al. [45]	Strong desynchronisation of occipital and temporal alpha band during the aura period, terminating abruptly upon disappearance of scintillations.	Analysis of MEG activity during an episode of scintillating scotoma	Patient with aura
Bjørk and Sand [46]	Increased occipital alpha power during the attack phase.	5 min eyes-closed EEG-videometry	41 migraine patients (33 MO, 8 MA)
Bjørk et al. [47]	Alpha peak frequency reduction correlated with increasing disease- and attack-duration. Frequency variability increased before the attack, while peak alpha power increased during the attack.	5 min eyes-closed EEG-videometry	41 migraine patients (33 MO, 8 MA) and 32 controls
Clemens et al. [49]	Increased alpha power in the migraineurs than in the control group in the right occipital region.	Resting-state EEG	20 MO patients and 17 controls.
Lia et al. [50]	Increased relative alpha power in migraineurs, particularly in posterior regions.	Resting-state EEG	17 MO and 11 MA patients and 28 controls.
Gomez-Pilar et al. [51]	Difference in the upper-alpha band in migraineurs, with differences evident in the central and left parietal regions.	Resting-state EEG	87 patients with migraine (45 with episodic and 42 with chronic migraine) and 39 controls.
O’Hare et al. [52]	Increased occipital resting-state alpha power in the mixed migraine group compared to controls.	Resting-state EEG	13 patients with migraine and 17 controls
Neufeld et al. [61]	Faster peak alpha frequency over posterior areas in migraineurs compared to control groups. The peak alpha power was lower among patients than controls.	Resting-state EEG	42 migraineurs and 20 controls.
Bjørk et al. [62]	Absolute power and alpha peak frequency were similar between groups.	EEG with photic stimulation on different days.	41 migraineurs (33 MO, 8 MA), 32 controls.
Fong et al. [69]	Migraineurs had significantly less posterior alpha power prior to the onset of the stimulus relative to controls. Migraineurs showed greater poststimulus alpha desynchronisation.	EEG activity during visual stimulation	28 migraineurs (11 MO, 17 MA) and 29 controls.
de Tommaso et al. [73]	Increased alpha synchronisation in migraineurs compared to controls using flash stimulation.	EEG activity during flash stimuli presentation	45 MO patients and 24 controls.

**Table 2:** A summary of the main MEG/EEG studies showing alteration in gamma oscillations in migraine.

Study	Summary of Findings	Paradigm	Sample
Hall et al. [45]	Strong gamma-band desynchronisation in temporal areas for the 1 min of the migraine aura, slowly returning to baseline levels over a 16-min period.	Analysis of MEG activity during an episode of scintillating scotoma	Patient with aura
Liu et al. [94]	Increase in gamma power in the lateral cortical regions in those with acute migraine (both MA and MO) compared to controls.	Analysis of MEG activity during the headache attack.	22 patients with an acute migraine and 22 controls.
Li et al. [95]	Gamma-band oscillations in left frontal and temporal areas have higher power in migraineurs compared to control groups.	Resting-state MEG	25 migraine patients during the headache-free phase and 25 controls.
Bassez et al. [96]	No difference between controls and MO groups in the gamma-band response to painful laser stimulation.	EEG during laser stimulation	23 MO patients and 23 controls
Coppola et al. [28]	Evoked gamma-band amplitude recorded over the occipital region is increased in between attacks in MA.	EEG during visual stimulation	15 MO and 15 MA patients
Lisicki et al. [106]	Ictal and chronic migraine patients showed increased gamma-band power.	EEG during visual stimulation	70 migraine patients (30 interictal, 20 ictal episodic migraineurs, 20 chronic migraineurs), and 20 controls.
Coppola et al. [107]	Early high-frequency oscillations were smaller in the mixed migraine (MO and MA) during the interictal phase compared to control group	EEG activity of parietal area during somatosensory stimulation	29 migraineurs (14 MO, 15 MA) during the interictal phase, 13 migraineurs (9 MO, 4 MA) during the ictal phase, and 15 controls.

**Table 3:** A summary of the main studies showing alteration in glutamate in migraine.

Study	Summary of Findings	Paradigm	Sample
Prescot et al. [124]	No differences in glutamate levels between migraineurs and control participants in the anterior cingulate cortex and insula.	MRS	10 migraine patients, and 8 controls
Gonzalez de la Aleja et al. [127]	Higher glutamate levels in migraineurs. Higher glutamate/glutamine ratio in the occipital cortex of migraineurs compared with controls.	MRS	27 patients with migraine (19 MO, 8 MA) and 19 controls.
Bathel et al. [128]	Increased Glx concentration in the thalamus and occipital regions in MO.	MRS	15 MO patients and 15 controls.
Siniatchkin et al. [129]	Migraineurs showed significantly higher glutamate/creatine ratios (Glx/Cr).	MRS	10 MA patients and 10 controls.
Zielman et al. [130]	Higher glutamate concentration in MO compared to controls, but not MA compared to controls	MRS	63 patients with migraine (36 MO, 27 MA) and 27 controls.
Wang et al. [131]	Higher Glx/water ratios in chronic but not episodic migraine in the periaqueductal gray.	MRS	25 chronic migraine patients, 24 episodic migraine patients and 16 controls.
Niddam et al. [132]	No group differences in the concentration of glutamate and glutamine.	MRS	25 chronic migraine patients, 24 episodic migraine patients and 25 controls.
Peek et al. [133]	Higher levels of glutamate in migraineurs compared to controls.	Meta-Analysis	35 studies were included investigating combinations of migraine (n = 11), musculoskeletal pain (n = 8), chronic pain syndromes (n = 9) and miscellaneous pain (n = 10).
Bridge et al. [134]	Glutamate levels in migraineurs, but not controls, correlated with BOLD signal in the primary visual cortex during visual stimulation.	MRS	13 MA patients and 13 controls.

**Table 4:** A summary of the main studies showing alteration in GABA in migraine.

Study	Summary of Findings	Paradigm	Sample
Bridge et al. [134]	GABA levels in the occipital cortex were lower in migraineurs than controls.	MRS	13 MA patients and 13 controls.
Bigal et al. [138]	GABA levels were not significantly different in migraineurs and controls. When pooling the MO and MA patients, GABA concentration was lower in individuals with severe headaches in the previous month.	MRS	9 MA patients, 10 MO patients, and 9 controls.
Onderwater et al. [139]	GABA levels increased from interictal towards the preictal state in migraineurs compared with controls in a provoked migraine attack.	MRS	24 MO patients and 13 controls.
Wang et al. [131]	Chronic migraineurs had significantly lower levels of GABA/water and GABA/creatine in the dentate nucleus.	MRS	25 chronic migraine patients, 24 episodic migraine patients, and 16 controls.
Wu et al. [140]	Lower GABA concentration in the anterior cingulate cortex and medial prefrontal lobe in migraineurs than controls.	MRS	28 MO patients and 28 controls.
Chan et al. [141]	Occipital GABA levels were similar between groups.	MRS	9 MA patients, 7 MO patients, and 16 controls.
Zhang et al. [147]	Lower GABA concentration in chronic migraine.GABA/Glx ratio was lower in the chronic than in the episodic group.	MRS	26 patients (episodic migraine = 11; chronic migraine = 15) and 16 controls.
Chan et al. [142]	No significant difference in GABA and glutamate levels were found between groups.	MRS	18 patients with migraine and 18 controls
Vieira et al. [145]	Chronic migraineurs with comorbid depression showed reduced GABA levels.	Cerebrospinal fluid (CSF) analysis	14 chronic migraine patients, with or without depression and 14 controls.
Strmose et al. [143]	No difference was found in GABA/total creatine levels in either the occipital cortex or in the somatosensory cortex.	MRS	14 MA patients and 16 controls.

## Data Availability

Not applicable.

## Abbreviations

The following abbreviations are used in this manuscript:

CSD	Cortical spreading depression
GABA	Gamma-aminobutyric acid
Glx	Glutamate + glutamine
MO	Migraine without aura
MA	Migraine with aura
PET	Positron emission tomography
SAI	Short latency afferent inhibition
SSVEP	Steady-state visual evoked potential
tACS	Transcranial alternating current stimulation
tDCA	Transcranial direct current stimulation
tES	Transcranial electrical stimulation
TMS	Transcranial magnetic stimulation

## References

[1] Woldeamanuel Y.W., Cowan R.P. (2017). Migraine affects 1 in 10 people worldwide featuring recent rise: A systematic review and meta-analysis of community-based studies involving 6 million participants. J. Neurol. Sci..

[2] Ferrari M.D. (1998). The economic burden of migraine to society. Pharmacoeconomics.

[3] Headache Classification Committee (HCC) of the International Headache Society (IHS) (2018). The International Classification of Headache Disorders, 3rd edition. Cephalalgia.

[4] Bigal M.E., Liberman J.N., Lipton R.B. (2006). Age-dependent prevalence and clinical features of migraine. Neurology.

[5] Lipton R.B., Stewart W.F., Diamond S., Diamond M.L., Reed M. (2001). Prevalence and burden of migraine in the United States: Data from the American Migraine Study II. Headache J. Head Face Pain.

[6] Lyngberg A.C., Rasmussen B.K., Jørgensen T., Jensen R. (2005). Prognosis of migraine and tension-type headache: A population-based follow-up study. Neurology.

[7] Jen J.C., Adam M.P., Mirzaa G.M., Pagon R.A., Wallace S.E., Bean L.J.H., Gripp K.W., Amemiya A. (2001). Familial Hemiplegic Migraine. GeneReviews®.

[8] Schott G.D. (2007). Exploring the visual hallucinations of migraine aura: The tacit contribution of illustration. Brain.

[9] O’Hare L., Asher J.M., Hibbard P.B. (2021). Migraine Visual Aura and Cortical Spreading Depression—Linking Mathematical Models to Empirical Evidence. Vision.

[10] Hadjikhani N., Sanchez del Rio M., Wu O., Schwartz D., Bakker D., Fischl B., Kwong K.K., Cutrer F.M., Rosen B.R., Tootell R.B. (2001). Mechanisms of migraine aura revealed by functional MRI in human visual cortex. Proc. Natl. Acad. Sci. USA.

[11] Borgdorff P. (2018). Arguments against the role of cortical spreading depression in migraine. Neurol. Res..

[12] Cutrer F.M., Charles A. (2008). The neurogenic basis of migraine. Headache J. Head Face Pain.

[13] Karsan N., Goadsby P.J. (2018). Biological insights from the premonitory symptoms of migraine. Nat. Rev. Neurol..

[14] Goadsby P.J., Holland P.R., Martins-Oliveira M., Hoffmann J., Schankin C., Akerman S. (2017). Pathophysiology of migraine: A disorder of sensory processing. Physiol. Rev..

[15] Wu Y., Hallett M. (2017). Photophobia in neurologic disorders. Transl. Neurodegener..

[16] Marcus D.A., Soso M.J. (1989). Migraine and stripe-induced visual discomfort. Arch. Neurol..

[17] Harle D.E., Shepherd A.J., Evans B.J. (2006). Visual stimuli are common triggers of migraine and are associated with pattern glare. Headache J. Head Face Pain.

[18] Pietrobon D. (2005). Migraine: New molecular mechanisms. Neuroscientist.

[19] Bigal M.E., Lipton R.B. (2008). Concepts and mechanisms of migraine chronification. Headache J. Head Face Pain.

[20] Bille B. (1997). A 40-year follow-up of school children with migraine. Cephalalgia.

[21] Chronicle E., Mulleners W. (1994). Might migraine damage the brain?. Cephalalgia.

[22] Buse D.C., Greisman J.D., Baigi K., Lipton R.B. (2019). Migraine progression: A systematic review. Headache J. Head Face Pain.

[23] Hernandez-Latorre M., Roig M. (2000). Natural history of migraine in childhood. Cephalalgia.

[24] Pietrobon D., Moskowitz M.A. (2013). Pathophysiology of migraine. Annu. Rev. Physiol..

[25] Coppola G., Pierelli F., Schoenen J. (2007). Is the cerebral cortex hyperexcitable or hyperresponsive in migraine?. Cephalalgia.

[26] Aguggia M., Saracco M. (2010). Pathophysiology of migraine chronification. Neurol. Sci..

[27] Aurora S., Wilkinson F. (2007). The brain is hyperexcitable in migraine. Cephalalgia.

[28] Coppola G., Ambrosini A., Clemente L.D., Magis D., Fumal A., Gerard P., Pierelli F., Schoenen J. (2007). Interictal abnormalities of gamma band activity in visual evoked responses in migraine: An indication of thalamocortical dysrhythmia?. Cephalalgia.

[29] Roux F., Wibral M., Singer W., Aru J., Uhlhaas P.J. (2013). The phase of thalamic alpha activity modulates cortical gamma-band activity: Evidence from resting-state MEG recordings. J. Neurosci..

[30] Rowley N.M., Madsen K.K., Schousboe A., White H.S. (2012). Glutamate and GABA synthesis, release, transport and metabolism as targets for seizure control. Neurochem. Int..

[31] Kossoff E.H., Andermann F. (2010). Migraine and epilepsy. Proceedings of the Seminars in Pediatric Neurology.

[32] Peek A.L., Leaver A.M., Foster S., Oeltzschner G., Puts N.A., Galloway G., Sterling M., Ng K., Refshauge K., Aguila M.E.R. (2021). Increased GABA+ in people with migraine, headache, and pain conditions-a potential marker of pain. J. Pain.

[33] Klimesch W. (1999). EEG alpha and theta oscillations reflect cognitive and memory performance: A review and analysis. Brain Res. Rev..

[34] Clayton M.S., Yeung N., Cohen Kadosh R. (2018). The effects of 10 Hz transcranial alternating current stimulation on audiovisual task switching. Front. Neurosci..

[35] Romei V., Rihs T., Brodbeck V., Thut G. (2008). Resting electroencephalogram alpha-power over posterior sites indexes baseline visual cortex excitability. Neuroreport.

[36] Romei V., Gross J., Thut G. (2010). On the role of prestimulus alpha rhythms over occipito-parietal areas in visual input regulation: Correlation or causation?. J. Neurosci..

[37] Romei V., Brodbeck V., Michel C., Amedi A., Pascual-Leone A., Thut G. (2008). Spontaneous fluctuations in posterior *α*-band EEG activity reflect variability in excitability of human visual areas. Cereb. Cortex.

[38] Tarasi L., Trajkovic J., Diciotti S., di Pellegrino G., Ferri F., Ursino M., Romei V. (2022). Predictive waves in the autism-schizophrenia continuum: A novel biobehavioral model. Neurosci. Biobehav. Rev..

[39] Ippolito G., Bertaccini R., Tarasi L., Di Gregorio F., Trajkovic J., Battaglia S., Romei V. (2022). The Role of Alpha Oscillations among the Main Neuropsychiatric Disorders in the Adult and Developing Human Brain: Evidence from the Last 10 Years of Research. Biomedicines.

[40] Stroganova T.A., Orekhova E.V., Posikera I.N. (1999). EEG alpha rhythm in infants. Clin. Neurophysiol..

[41] Jensen O., Mazaheri A. (2010). Shaping functional architecture by oscillatory alpha activity: Gating by inhibition. Front. Hum. Neurosci..

[42] Van Dijk H., Schoffelen J.M., Oostenveld R., Jensen O. (2008). Prestimulus oscillatory activity in the alpha band predicts visual discrimination ability. J. Neurosci..

[43] Limbach K., Corballis P.M. (2016). Prestimulus alpha power influences response criterion in a detection task. Psychophysiology.

[44] Tarasi L., di Pellegrino G., Romei V. (2022). Are you an empiricist or a believer? Neural signatures of predictive strategies in humans. Prog. Neurobiol..

[45] Hall S.D., Barnes G.R., Hillebrand A., Furlong P.L., Singh K.D., Holliday I.E. (2004). Spatio-temporal imaging of cortical desynchronization in migraine visual aura: A magnetoencephalography case study. Headache J. Head Face Pain.

[46] Bjørk M., Sand T. (2008). Quantitative EEG power and asymmetry increase 36 h before a migraine attack. Cephalalgia.

[47] Bjørk M., Stovner L., Nilsen B., Stjern M., Hagen K., Sand T. (2009). The occipital alpha rhythm related to the “migraine cycle” and headache burden: A blinded, controlled longitudinal study. Clin. Neurophysiol..

[48] Cao Z., Lin C.T., Chuang C.H., Lai K.L., Yang A.C., Fuh J.L., Wang S.J. (2016). Resting-state EEG power and coherence vary between migraine phases. J. Headache Pain.

[49] Clemens B., Bánk J., Piros P., Bessenyei M., Vető S., Tóth M., Kondákor I. (2008). Three-dimensional localization of abnormal EEG activity in migraine: A low resolution electromagnetic tomography (LORETA) study of migraine patients in the pain-free interval. Brain Topogr..

[50] Lia C., Carenini L., Degioz C., Bottachi E. (1995). Computerized EEG analysis in migraine patients. Ital. J. Neurol. Sci..

[51] Gomez-Pilar J., García-Azorín D., Gomez-Lopez-de San-Roman C., Guerrero Á.L., Hornero R. (2020). Exploring EEG spectral patterns in episodic and chronic migraine during the interictal state: Determining frequencies of interest in the resting state. Pain Med..

[52] O’Hare L., Menchinelli F., Durrant S.J. (2018). Resting-state alpha-band oscillations in migraine. Perception.

[53] Van Beijsterveldt C., Van Baal G. (2002). Twin and family studies of the human electroencephalogram: A review and a meta-analysis. Biol. Psychol..

[54] Angelakis E., Lubar J.F., Stathopoulou S., Kounios J. (2004). Peak alpha frequency: An electroencephalographic measure of cognitive preparedness. Clin. Neurophysiol..

[55] Klimesch W. (1997). EEG-alpha rhythms and memory processes. Int. J. Psychophysiol..

[56] Grandy T.H., Werkle-Bergner M., Chicherio C., Schmiedek F., Lövdén M., Lindenberger U. (2013). Peak individual alpha frequency qualifies as a stable neurophysiological trait marker in healthy younger and older adults. Psychophysiology.

[57] Zhang Y., Lu Y., Wang D., Zhou C., Xu C. (2021). Relationship between individual alpha peak frequency and attentional performance in a multiple object tracking task among ice-hockey players. PLoS ONE.

[58] Samaha J., Postle B.R. (2015). The speed of alpha-band oscillations predicts the temporal resolution of visual perception. Curr. Biol..

[59] Di Gregorio F., Trajkovic J., Roperti C., Marcantoni E., Di Luzio P., Avenanti A., Thut G., Romei V. (2022). Tuning alpha rhythms to shape conscious visual perception. Curr. Biol..

[60] Cecere R., Rees G., Romei V. (2015). Individual differences in alpha frequency drive crossmodal illusory perception. Curr. Biol..

[61] Neufeld M., Treves T., Korczyn A. (1991). EEG and topographic frequency analysis in common and classic migraine. Headache J. Head Face Pain.

[62] Bjørk M., Stovner L., Hagen K., Sand T. (2011). What initiates a migraine attack? Conclusions from four longitudinal studies of quantitative EEG and steady-state visual-evoked potentials in migraineurs. Acta Neurol. Scand..

[63] Furman A.J., Prokhorenko M., Keaser M.L., Zhang J., Chen S., Mazaheri A., Seminowicz D.A. (2020). Sensorimotor peak alpha frequency is a reliable biomarker of prolonged pain sensitivity. Cereb. Cortex.

[64] Valentini E., Halder S., McInnerney D., Cooke J., Gyimes I.L., Romei V. (2022). Assessing the specificity of the relationship between brain alpha oscillations and tonic pain. NeuroImage.

[65] Freschl J., Al Azizi L., Balboa L., Kaldy Z., Blaser E. (2022). The development of peak alpha frequency from infancy to adolescence and its role in visual temporal processing: A meta-analysis. Dev. Cogn. Neurosci..

[66] Clayton M.S., Yeung N., Cohen Kadosh R. (2018). The many characters of visual alpha oscillations. Eur. J. Neurosci..

[67] Lorincz M.L., Kékesi K.A., Juhász G., Crunelli V., Hughes S.W. (2009). Temporal framing of thalamic relay-mode firing by phasic inhibition during the alpha rhythm. Neuron.

[68] Nyrke T., Kangasniemi P., Lang H. (1990). Alpha rhythm in classical migraine (migraine with aura): Abnormalities in the headache-free interval. Cephalalgia.

[69] Fong C.Y., Law W.H.C., Fahrenfort J.J., Braithwaite J.J., Mazaheri A. (2022). Attenuated alpha oscillation and hyperresponsiveness reveals impaired perceptual learning in migraineurs. J. Headache Pain.

[70] Angelini L., De Tommaso M., Guido M., Hu K., Ivanov P.C., Marinazzo D., Nardulli G., Nitti L., Pellicoro M., Pierro C. (2004). Steady-state visual evoked potentials and phase synchronization in migraine patients. Phys. Rev. Lett..

[71] De Tommaso M., Stramaglia S., Marinazzo D., Guido M., Lamberti P., Livrea P. (2004). Visually evoked phase synchronisation changes of alpha rhythm in migraine. Correlations with clinical features. Neurol. Sci..

[72] De Tommaso M., Marinazzo D., Guido M., Libro G., Stramaglia S., Nitti L., Lattanzi G., Angelini L., Pellicoro M. (2005). Visually evoked phase synchronization changes of alpha rhythm in migraine: Correlations with clinical features. Int. J. Psychophysiol..

[73] De Tommaso M., Marinazzo D., Nitti L., Pellicoro M., Guido M., Serpino C., Stramaglia S. (2007). Effects of levetiracetam vs topiramate and placebo on visually evoked phase synchronization changes of alpha rhythm in migraine. Clin. Neurophysiol..

[74] De Tommaso M., Stramaglia S., Brighina F., Fierro B., Francesco V.D., Todarello O., Serpino C., Pellicoro M. (2011). Lack of effects of low frequency repetitive transcranial magnetic stimulation on alpha rhythm phase synchronization in migraine patients. Neurosci. Lett..

[75] Surges R., Volynski K.E., Walker M.C. (2008). Is levetiracetam different from other antiepileptic drugs? Levetiracetam and its cellular mechanism of action in epilepsy revisited. Ther. Adv. Neurol. Disord..

[76] Boroojerdi B., Prager A., Muellbacher W., Cohen L.G. (2000). Reduction of human visual cortex excitability using 1-Hz transcranial magnetic stimulation. Neurology.

[77] Caparelli E., Backus W., Telang F., Wang G., Maloney T., Goldstein R., Henn F. (2012). Is 1 Hz rTMS always inhibitory in healthy individuals?. Open Neuroimaging J..

[78] Fierro B., Ricci R., Piazza A., Scalia S., Giglia G., Vitello G., Brighina F. (2003). 1 Hz rTMS enhances extrastriate cortex activity in migraine: Evidence of a reduced inhibition?. Neurology.

[79] De Tommaso M., Stramaglia S., Marinazzo D., Trotta G., Pellicoro M. (2013). Functional and effective connectivity in EEG alpha and beta bands during intermittent flash stimulation in migraine with and without aura. Cephalalgia.

[80] Chamanzar A., Haigh S.M., Grover P., Behrmann M. (2021). Abnormalities in cortical pattern of coherence in migraine detected using ultra high-density EEG. Brain Commun..

[81] Hansen J.M., Charles A. (2019). Differences in treatment response between migraine with aura and migraine without aura: Lessons from clinical practice and RCTs. J. Headache Pain.

[82] Bertrand O., Tallon-Baudry C. (2000). Oscillatory gamma activity in humans: A possible role for object representation. Int. J. Psychophysiol..

[83] Van Pelt S., Boomsma D.I., Fries P. (2012). Magnetoencephalography in twins reveals a strong genetic determination of the peak frequency of visually induced gamma-band synchronization. J. Neurosci..

[84] Guan A., Wang S., Huang A., Qiu C., Li Y., Li X., Wang J., Wang Q., Deng B. (2022). The role of gamma oscillations in central nervous system diseases: Mechanism and treatment. Front. Cell. Neurosci..

[85] Csibra G., Davis G., Spratling M., Johnson M. (2000). Gamma oscillations and object processing in the infant brain. Science.

[86] Kessler K., Seymour R.A., Rippon G. (2016). Brain oscillations and connectivity in autism spectrum disorders (ASD): New approaches to methodology, measurement and modelling. Neurosci. Biobehav. Rev..

[87] Ren L., Kucewicz M.T., Cimbalnik J., Matsumoto J.Y., Brinkmann B.H., Hu W., Marsh W.R., Meyer F.B., Stead S.M., Worrell G.A. (2015). Gamma oscillations precede interictal epileptiform spikes in the seizure onset zone. Neurology.

[88] Tallon-Baudry C., Bertrand O. (1999). Oscillatory gamma activity in humans and its role in object representation. Trends Cogn. Sci..

[89] Hoogenboom N., Schoffelen J.M., Oostenveld R., Parkes L.M., Fries P. (2006). Localizing human visual gamma-band activity in frequency, time and space. Neuroimage.

[90] Steriade M., Contreras D., Amzica F., Timofeev I. (1996). Synchronization of fast (30-40 Hz) spontaneous oscillations in intrathalamic and thalamocortical networks. J. Neurosci..

[91] Buchner H., Gobbele R., Waberski T.D., Wagner M., Fuchs M. (1999). Evidence for independent thalamic and cortical sources involved in the generation of the visual 40 Hz response in humans. Neurosci. Lett..

[92] Orekhova E.V., Sysoeva O.V., Schneiderman J.F., Lundström S., Galuta I.A., Goiaeva D.E., Prokofyev A.O., Riaz B., Keeler C., Hadjikhani N. (2018). Input-dependent modulation of MEG gamma oscillations reflects gain control in the visual cortex. Sci. Rep..

[93] Orekhova E.V., Stroganova T.A., Schneiderman J.F., Lundström S., Riaz B., Sarovic D., Sysoeva O.V., Brant G., Gillberg C., Hadjikhani N. (2019). Neural gain control measured through cortical gamma oscillations is associated with sensory sensitivity. Hum. Brain Mapp..

[94] Liu H., Ge H., Xiang J., Miao A., Tang L., Wu T., Chen Q., Yang L., Wang X. (2015). Resting state brain activity in patients with migraine: A magnetoencephalography study. J. Headache Pain.

[95] Li F., Xiang J., Wu T., Zhu D., Shi J. (2016). Abnormal resting-state brain activity in headache-free migraine patients: A magnetoencephalography study. Clin. Neurophysiol..

[96] Bassez I., Ricci K., Vecchio E., Delussi M., Gentile E., Marinazzo D., de Tommaso M. (2020). The effect of painful laser stimuli on EEG gamma-band activity in migraine patients and healthy controls. Clin. Neurophysiol..

[97] Ren J., Xiang J., Chen Y., Li F., Wu T., Shi J. (2019). Abnormal functional connectivity under somatosensory stimulation in migraine: A multi-frequency magnetoencephalography study. J. Headache Pain.

[98] Barlow H.B. (1961). Possible principles underlying the transformation of sensory messages. Sens. Commun..

[99] Hibbard P.B., O’Hare L. (2015). Uncomfortable images produce non-sparse responses in a model of primary visual cortex. R. Soc. Open Sci..

[100] O’Hare L., Clarke A.D., Pollux P.M. (2015). VEP responses to op-art stimuli. PLoS ONE.

[101] O’Hare L. (2017). Steady-state VEP responses to uncomfortable stimuli. Eur. J. Neurosci..

[102] O’Hare L., Hird E., Whybrow M. (2021). Steady-state visual evoked potential responses predict visual discomfort judgements. Eur. J. Neurosci..

[103] Vanagaite J., Pareja J., Støren O., White L., Sanc T., Stovner L. (1997). Light-induced discomfort and pain in migraine. Cephalalgia.

[104] Shepherd A.J., Hine T.J., Beaumont H.M. (2013). Color and spatial frequency are related to visual pattern sensitivity in migraine. Headache J. Head Face Pain.

[105] Orekhova E.V., Rostovtseva E.N., Manyukhina V.O., Prokofiev A.O., Obukhova T.S., Nikolaeva A.Y., Schneiderman J.F., Stroganova T.A. (2020). Spatial suppression in visual motion perception is driven by inhibition: Evidence from MEG gamma oscillations. NeuroImage.

[106] Lisicki M., D’Ostilio K., Coppola G., Nonis R., de Noordhout A.M., Parisi V., Magis D., Schoenen J. (2020). Headache related alterations of visual processing in migraine patients. J. Pain.

[107] Coppola G., Vandenheede M., Di Clemente L., Ambrosini A., Fumal A., De Pasqua V., Schoenen J. (2005). Somatosensory evoked high-frequency oscillations reflecting thalamo-cortical activity are decreased in migraine patients between attacks. Brain.

[108] Schroeder C.E., Lakatos P. (2009). Low-frequency neuronal oscillations as instruments of sensory selection. Trends Neurosci..

[109] Osipova D., Hermes D., Jensen O. (2008). Gamma power is phase-locked to posterior alpha activity. PLoS ONE.

[110] Herring J.D., Esterer S., Marshall T.R., Jensen O., Bergmann T.O. (2019). Low-frequency alternating current stimulation rhythmically suppresses gamma-band oscillations and impairs perceptual performance. Neuroimage.

[111] Orekhova E.V., Prokofyev A.O., Nikolaeva A.Y., Schneiderman J.F., Stroganova T.A. (2020). Additive effect of contrast and velocity suggests the role of strong excitatory drive in suppression of visual gamma response. PLoS ONE.

[112] Jensen O., Colgin L.L. (2007). Cross-frequency coupling between neuronal oscillations. Trends Cogn. Sci..

[113] Llinás R.R., Ribary U., Jeanmonod D., Kronberg E., Mitra P.P. (1999). Thalamocortical dysrhythmia: A neurological and neuropsychiatric syndrome characterized by magnetoencephalography. Proc. Natl. Acad. Sci. USA.

[114] Schreckenberger M., Lange-Asschenfeld C., Lochmann M., Mann K., Siessmeier T., Buchholz H.G., Bartenstein P., Gründer G. (2004). The thalamus as the generator and modulator of EEG alpha rhythm: A combined PET/EEG study with lorazepam challenge in humans. Neuroimage.

[115] Coppola G., Bracaglia M., Di Lenola D., Iacovelli E., Di Lorenzo C., Serrao M., Evangelista M., Parisi V., Schoenen J., Pierelli F. (2016). Lateral inhibition in the somatosensory cortex during and between migraine without aura attacks: Correlations with thalamocortical activity and clinical features. Cephalalgia.

[116] Hodkinson D.J., Wilcox S.L., Veggeberg R., Noseda R., Burstein R., Borsook D., Becerra L. (2016). Increased amplitude of thalamocortical low-frequency oscillations in patients with migraine. J. Neurosci..

[117] Tu Y., Fu Z., Zeng F., Maleki N., Lan L., Li Z., Park J., Wilson G., Gao Y., Liu M. (2019). Abnormal thalamocortical network dynamics in migraine. Neurology.

[118] Raichle M.E., MacLeod A.M., Snyder A.Z., Powers W.J., Gusnard D.A., Shulman G.L. (2001). A default mode of brain function. Proc. Natl. Acad. Sci. USA.

[119] Martins I.P., Westerfield M., Lopes M., Maruta C., Gil-da Costa R. (2020). Brain state monitoring for the future prediction of migraine attacks. Cephalalgia.

[120] Coppola G., Di Renzo A., Tinelli E., Di Lorenzo C., Di Lorenzo G., Parisi V., Serrao M., Schoenen J., Pierelli F. (2016). Thalamo-cortical network activity during spontaneous migraine attacks. Neurology.

[121] Coppola G., Di Lenola D., Abagnale C., Ferrandes F., Sebastianelli G., Casillo F., Di Lorenzo C., Serrao M., Evangelista M., Schoenen J. (2020). Short-latency afferent inhibition and somato-sensory evoked potentials during the migraine cycle: Surrogate markers of a cycling cholinergic thalamo-cortical drive?. J. Headache Pain.

[122] Martinelli D., Castellazzi G., De Icco R., Bacila A., Allena M., Faggioli A., Sances G., Pichiecchio A., Borsook D., Gandini Wheeler-Kingshott C.A. (2021). Thalamocortical connectivity in experimentally-induced migraine attacks: A pilot study. Brain Sci..

[123] Meneghetti N., Cerri C., Vannini E., Tantillo E., Tottene A., Pietrobon D., Caleo M., Mazzoni A. (2022). Synaptic alterations in visual cortex reshape contrast-dependent gamma oscillations and inhibition-excitation ratio in a genetic mouse model of migraine. J. Headache Pain.

[124] Prescot A., Becerra L., Pendse G., Tully S., Jensen E., Hargreaves R., Renshaw P., Burstein R., Borsook D. (2009). Excitatory neurotransmitters in brain regions in interictal migraine patients. Mol. Pain.

[125] Arngrim N., Schytz H.W., Britze J., Amin F.M., Vestergaard M.B., Hougaard A., Wolfram F., de Koning P.J., Olsen K.S., Secher N.H. (2016). Migraine induced by hypoxia: An MRI spectroscopy and angiography study. Brain.

[126] Petrusic I., Zidverc-Trajkovic J. (2014). Cortical spreading depression: Origins and paths as inferred from the sequence of events during migraine aura. Funct. Neurol..

[127] Gonzalez de la Aleja J., Ramos A., Mato-Abad V., Martínez-Salio A., Hernández-Tamames J.A., Molina J.A., Hernández-Gallego J., Álvarez-Linera J. (2013). Higher glutamate to glutamine ratios in occipital regions in women with migraine during the interictal state. Headache J. Head Face Pain.

[128] Bathel A., Schweizer L., Stude P., Glaubitz B., Wulms N., Delice S., Schmidt-Wilcke T. (2018). Increased thalamic glutamate/glutamine levels in migraineurs. J. Headache Pain.

[129] Siniatchkin M., Sendacki M., Moeller F., Wolff S., Jansen O., Siebner H., Stephani U. (2012). Abnormal changes of synaptic excitability in migraine with aura. Cereb. Cortex.

[130] Zielman R., Wijnen J.P., Webb A., Onderwater G.L., Ronen I., Ferrari M.D., Kan H.E., Terwindt G.M., Kruit M.C. (2017). Cortical glutamate in migraine. Brain.

[131] Wang W., Zhang X., Bai X., Zhang Y., Yuan Z., Tang H., Li Z., Hu Z., Zhang Y., Yu X. (2022). Gamma-aminobutyric acid and glutamate/glutamine levels in the dentate nucleus and periaqueductal gray with episodic and chronic migraine: A proton magnetic resonance spectroscopy study. J. Headache Pain.

[132] Niddam D.M., Lai K.L., Tsai S.Y., Lin Y.R., Chen W.T., Fuh J.L., Wang S.J. (2018). Neurochemical changes in the medial wall of the brain in chronic migraine. Brain.

[133] Peek A.L., Rebbeck T., Puts N.A., Watson J., Aguila M.E.R., Leaver A.M. (2020). Brain GABA and glutamate levels across pain conditions: A systematic literature review and meta-analysis of 1H-MRS studies using the MRS-Q quality assessment tool. Neuroimage.

[134] Bridge H., Stagg C.J., Near J., Lau C.i., Zisner A., Cader M.Z. (2015). Altered neurochemical coupling in the occipital cortex in migraine with visual aura. Cephalalgia.

[135] Fayed N., Andrés E., Viguera L., Modrego P.J., Garcia-Campayo J. (2014). Higher glutamate+ glutamine and reduction of N-acetylaspartate in posterior cingulate according to age range in patients with cognitive impairment and/or pain. Acad. Radiol..

[136] Perenboom M., Najafabadi A.Z., Zielman R., Tolner E., Carpay J., Ferrari M. (2015). Visual sensitivity is more enhanced in migraineurs with aura than in migraineurs without aura. Cephalalgia.

[137] Evans B., Stevenson S. (2008). The Pattern Glare Test: A review and determination of normative values. Ophthalmic Physiol. Opt..

[138] Bigal M., Hetherington H., Pan J., Tsang A., Grosberg B., Avdievich N., Friedman B., Lipton R. (2008). Occipital levels of GABA are related to severe headaches in migraine. Neurology.

[139] Onderwater G.L., Wijnen J.P., Najac C., van Dongen R.M., Ronen I., Webb A., Zielman R., van Zwet E.W., Ferrari M.D., Kan H.E. (2021). Cortical glutamate and gamma-aminobutyric acid over the course of a provoked migraine attack, a 7 Tesla magnetic resonance spectroscopy study. Neuroimage Clin..

[140] Wu X., Han S., Yang Y., Dai H., Wu P., Zhao H., Jin X., Li Y. (2022). Decreased brain GABA levels in patients with migraine without aura: An exploratory proton magnetic resonance spectroscopy study. Neuroscience.

[141] Chan Y.M., Pitchaimuthu K., Wu Q.Z., Carter O.L., Egan G.F., Badcock D.R., McKendrick A.M. (2019). Relating excitatory and inhibitory neurochemicals to visual perception: A magnetic resonance study of occipital cortex between migraine events. PLoS ONE.

[142] Chan Y.M., Glarin R., Moffat B.A., Bode S., McKendrick A.M. (2022). Relating the cortical visual contrast gain response to spectroscopy-measured excitatory and inhibitory metabolites in people who experience migraine. PLoS ONE.

[143] Stærmose T.G., Knudsen M.K., Kasch H., Blicher J.U. (2019). Cortical GABA in migraine with aura-an ultrashort echo magnetic resonance spectroscopy study. J. Headache Pain.

[144] Pohl H., Wyss P., Sandor P.S., Schoenen J., Luechinger R., O’Gorman R., Riederer F., Gantenbein A.R., Michels L. (2023). The longitudinal influence of tDCS on occipital GABA and glutamate/glutamine levels in episodic migraineurs. J. Neurosci. Res..

[145] Vieira D., Naffah-Mazacoratti M., Zukerman E., Soares C.S., Alonso E., Faulhaber M., Cavalheiro E., Peres M. (2006). Cerebrospinal fluid GABA levels in chronic migraine with and without depression. Brain Res..

[146] Bell T., Stokoe M., Khaira A., Webb M., Noel M., Amoozegar F., Harris A.D. (2021). GABA and glutamate in pediatric migraine. Pain.

[147] Zhang X., Wang W., Bai X., Zhang Y., Yuan Z., Tang H., Zhang X., Li Z., Zhang P., Hu Z. (2023). Changes in gamma-aminobutyric acid and glutamate/glutamine levels in the right thalamus of patients with episodic and chronic migraine: A proton magnetic resonance spectroscopy study. Headache J. Head Face Pain.

[148] Mathers D.A., Wan X., Puil E. (2007). Barbiturate activation and modulation of GABAA receptors in neocortex. Neuropharmacology.

[149] Bigal M.E., Lipton R., Tepper S., Rapoport A., Sheftell F. (2004). Primary chronic daily headache and its subtypes in adolescents and adults. Neurology.

[150] Aguila M.E.R., Lagopoulos J., Leaver A.M., Rebbeck T., Hübscher M., Brennan P.C., Refshauge K.M. (2015). Elevated levels of GABA+ in migraine detected using 1H-MRS. NMR Biomed..

[151] Zhang L., Huang J., Zhang Z., Cao Z. (2021). Altered metabolites in the occipital lobe in migraine without aura during the attack and the interictal period. Front. Neurol..

[152] Peek A.L., Leaver A.M., Foster S., Puts N.A., Oeltzschner G., Henderson L., Galloway G., Ng K., Refshauge K., Rebbeck T. (2021). Increase in ACC GABA+ levels correlate with decrease in migraine frequency, intensity and disability over time. J. Headache Pain.

[153] Burch R. (2019). Antidepressants for preventive treatment of migraine. Curr. Treat. Options Neurol..

[154] Drummond P. (2006). Tryptophan depletion increases nausea, headache and photophobia in migraine sufferers. Cephalalgia.

[155] Razeghi Jahromi S., Togha M., Ghorbani Z., Hekmatdoost A., Khorsha F., Rafiee P., Shirani P., Nourmohammadi M., Ansari H. (2019). The association between dietary tryptophan intake and migraine. Neurol. Sci..

[156] Ferrari M., Odink J., Tapparelli C., Van Kempen G., Pennings E., Bruyn G. (1989). Serotonin metabolism in migraine. Neurology.

[157] Deen M., Correnti E., Kamm K., Kelderman T., Papetti L., Rubio-Beltrán E., Vigneri S., Edvinsson L., Maassen Van Den Brink A. (2017). Blocking CGRP in migraine patients–a review of pros and cons. J. Headache Pain.

[158] Shields K.G., Goadsby P.J. (2006). Serotonin receptors modulate trigeminovascular responses in ventroposteromedial nucleus of thalamus: A migraine target?. Neurobiol. Dis..

[159] Supornsilpchai W., Sanguanrangsirikul S., Maneesri S., Srikiatkhachorn A. (2006). Serotonin depletion, cortical spreading depression, and trigeminal nociception. Headache J. Head Face Pain.

[160] Jacobs G., Van der Grond J., Teeuwisse W., Langeveld T., Van Pelt J., Verhagen J., de Kam M., Cohen A., Zitman F., van Gerven J. (2010). Hypothalamic glutamate levels following serotonergic stimulation: A pilot study using 7-Tesla magnetic resonance spectroscopy in healthy volunteers. Prog. -Neuro-Psychopharmacol. Biol. Psychiatry.

[161] Sanacora G., Mason G.F., Rothman D.L., Krystal J.H. (2002). Increased occipital cortex GABA concentrations in depressed patients after therapy with selective serotonin reuptake inhibitors. Am. J. Psychiatry.

[162] Bhagwagar Z., Wylezinska M., Taylor M., Jezzard P., Matthews P.M., Cowen P.J. (2004). Increased brain GABA concentrations following acute administration of a selective serotonin reuptake inhibitor. Am. J. Psychiatry.

[163] Silva L.R., Amitai Y., Connors B.W. (1991). Intrinsic oscillations of neocortex generated by layer 5 pyramidal neurons. Science.

[164] Vijayan S., Kopell N.J. (2012). Thalamic model of awake alpha oscillations and implications for stimulus processing. Proc. Natl. Acad. Sci. USA.

[165] Moghaddam B., Adams B., Verma A., Daly D. (1997). Activation of glutamatergic neurotransmission by ketamine: A novel step in the pathway from NMDA receptor blockade to dopaminergic and cognitive disruptions associated with the prefrontal cortex. J. Neurosci..

[166] Muthukumaraswamy S.D., Shaw A.D., Jackson L.E., Hall J., Moran R., Saxena N. (2015). Evidence that subanesthetic doses of ketamine cause sustained disruptions of NMDA and AMPA-mediated frontoparietal connectivity in humans. J. Neurosci..

[167] De la Salle S., Choueiry J., Shah D., Bowers H., McIntosh J., Ilivitsky V., Knott V. (2016). Effects of ketamine on resting-state EEG activity and their relationship to perceptual/dissociative symptoms in healthy humans. Front. Pharmacol..

[168] Lozano-Soldevilla D. (2018). On the physiological modulation and potential mechanisms underlying parieto-occipital alpha oscillations. Front. Comput. Neurosci..

[169] Lozano-Soldevilla D., ter Huurne N., Cools R., Jensen O. (2014). GABAergic modulation of visual gamma and alpha oscillations and its consequences for working memory performance. Curr. Biol..

[170] Baumgarten T.J., Neugebauer J., Oeltzschner G., Füllenbach N.D., Kircheis G., Häussinger D., Lange J., Wittsack H.J., Butz M., Schnitzler A. (2018). Connecting occipital alpha band peak frequency, visual temporal resolution, and occipital GABA levels in healthy participants and hepatic encephalopathy patients. Neuroimage Clin..

[171] Buzsáki G., Wang X.J. (2012). Mechanisms of gamma oscillations. Annu. Rev. Neurosci..

[172] Whittington M.A., Traub R.D., Jefferys J.G. (1995). Synchronized oscillations in interneuron networks driven by metabotropic glutamate receptor activation. Nature.

[173] Volman V., Behrens M.M., Sejnowski T.J. (2011). Downregulation of parvalbumin at cortical GABA synapses reduces network gamma oscillatory activity. J. Neurosci..

[174] Muthukumaraswamy S.D., Edden R.A., Jones D.K., Swettenham J.B., Singh K.D. (2009). Resting GABA concentration predicts peak gamma frequency and fMRI amplitude in response to visual stimulation in humans. Proc. Natl. Acad. Sci. USA.

[175] Gaetz W., Edgar J.C., Wang D., Roberts T.P. (2011). Relating MEG measured motor cortical oscillations to resting *γ*-aminobutyric acid (GABA) concentration. Neuroimage.

[176] Cousijn H., Haegens S., Wallis G., Near J., Stokes M.G., Harrison P.J., Nobre A.C. (2014). Resting GABA and glutamate concentrations do not predict visual gamma frequency or amplitude. Proc. Natl. Acad. Sci. USA.

[177] Wyss C., Tse D.H., Kometer M., Dammers J., Achermann R., Shah N.J., Kawohl W., Neuner I. (2017). GABA metabolism and its role in gamma-band oscillatory activity during auditory processing: An MRS and EEG study. Hum. Brain Mapp..

[178] Kujala J., Jung J., Bouvard S., Lecaignard F., Lothe A., Bouet R., Ciumas C., Ryvlin P., Jerbi K. (2015). Gamma oscillations in V1 are correlated with GABAA receptor density: A multi-modal MEG and Flumazenil-PET study. Sci. Rep..

[179] Storer R.J., Akerman S., Goadsby P.J. (2001). GABA receptors modulate trigeminovascular nociceptive neurotransmission in the trigeminocervical complex. Br. J. Pharmacol..

[180] Linde M., Mulleners W.M., Chronicle E.P., McCrory D.C. (2013). Valproate for preventing migraine attacks in adults. Cochrane Database Syst. Rev..

[181] Rad R.E., Ghaffari F., Fotokian Z., Ramezani A. (2017). The effectiveness of ibuprofen and lorazepam combination therapy in treating the symptoms of acute Migraine: A randomized clinical trial. Electron. Physician.

[182] Harnod T., Wang Y.C., Lin C.L., Tseng C.H. (2017). Association between use of short-acting benzodiazepines and migraine occurrence: A nationwide population-based case–control study. Curr. Med Res. Opin..

[183] Rivolta D., Heidegger T., Scheller B., Sauer A., Schaum M., Birkner K., Singer W., Wibral M., Uhlhaas P.J. (2015). Ketamine dysregulates the amplitude and connectivity of high-frequency oscillations in cortical–subcortical networks in humans: Evidence from resting-state magnetoencephalography-recordings. Schizophr. Bull..

[184] Lally N., Mullins P.G., Roberts M.V., Price D., Gruber T., Haenschel C. (2014). Glutamatergic correlates of gamma-band oscillatory activity during cognition: A concurrent ER-MRS and EEG study. Neuroimage.

[185] Nutt D., Wilson S., Lingford-Hughes A., Myers J., Papadopoulos A., Muthukumaraswamy S. (2015). Differences between magnetoencephalographic (MEG) spectral profiles of drugs acting on GABA at synaptic and extrasynaptic sites: A study in healthy volunteers. Neuropharmacology.

[186] Roberts E., Frankel S. (1950). *γ*-Aminobutyric acid in brain: Its formation from glutamic acid. J. Biol. Chem..

[187] Krause M.R., Vieira P.G., Pack C.C. (2023). Transcranial electrical stimulation: How can a simple conductor orchestrate complex brain activity?. PLoS Biol..

[188] Zhaoping L., May K.A. (2007). Psychophysical tests of the hypothesis of a bottom-up saliency map in primary visual cortex. PLoS Comput. Biol..

[189] Zhang X., Zhaoping L., Zhou T., Fang F. (2012). Neural activities in V1 create a bottom-up saliency map. Neuron.

[190] Glomb K., Cabral J., Cattani A., Mazzoni A., Raj A., Franceschiello B. (2021). Computational models in electroencephalography. Brain Topogr..

[191] Hodgkin A.L., Huxley A.F. (1952). A quantitative description of membrane current and its application to conduction and excitation in nerve. J. Physiol..

[192] Destexhe A., Bal T., McCormick D.A., Sejnowski T.J. (1996). Ionic mechanisms underlying synchronized oscillations and propagating waves in a model of ferret thalamic slices. J. Neurophysiol..

[193] Destexhe A., Sejnowski T.J. (1997). Synchronized oscillations in thalamic networks: Insights from modeling studies. Thalamus.

[194] Da Silva F.L., Van Leeuwen W.S. (1977). The cortical source of the alpha rhythm. Neurosci. Lett..

[195] Teleńczuk M., Teleńczuk B., Destexhe A. (2020). Modelling unitary fields and the single-neuron contribution to local field potentials in the hippocampus. J. Physiol..

[196] Wilson H.R., Cowan J.D. (1973). A mathematical theory of the functional dynamics of cortical and thalamic nervous tissue. Kybernetik.

[197] Börgers C., Börgers C. (2017). A Wilson-Cowan Model of an Oscillatory EI Network. An Introduction to Modeling Neuronal Dynamics.

[198] Bell T., Khaira A., Stokoe M., Webb M., Noel M., Amoozegar F., Harris A.D. (2021). Age-related differences in resting state functional connectivity in pediatric migraine. J. Headache Pain.

[199] Colon E., Ludwick A., Wilcox S.L., Youssef A.M., Danehy A., Fair D.A., Lebel A.A., Burstein R., Becerra L., Borsook D. (2019). Migraine in the young brain: Adolescents vs. young adults. Front. Hum. Neurosci..

[200] Messina R., Rocca M.A., Colombo B., Valsasina P., Meani A., Falini A., Filippi M. (2020). Dysregulation of multisensory processing stands out from an early stage of migraine: A study in pediatric patients. J. Neurol..

[201] Silvestro M., Tessitore A., Caiazzo G., Scotto di Clemente F., Trojsi F., Cirillo M., Esposito F., Tedeschi G., Russo A. (2021). Disconnectome of the migraine brain: A “connectopathy” model. J. Headache Pain.

[202] Klimesch W., Sauseng P., Hanslmayr S. (2007). EEG alpha oscillations: The inhibition–timing hypothesis. Brain Res. Rev..

[203] Steriade M., Gloor P., Llinas R.R., Da Silva F.L., Mesulam M.M. (1990). Basic mechanisms of cerebral rhythmic activities. Electroencephalogr. Clin. Neurophysiol..

[204] Da Silva F.L. (1991). Neural mechanisms underlying brain waves: From neural membranes to networks. Electroencephalogr. Clin. Neurophysiol..

[205] Castro-Alamancos M.A., Connors B.W. (1996). Cellular mechanisms of the augmenting response: Short-term plasticity in a thalamocortical pathway. J. Neurosci..

[206] Flint A.C., Connors B.W. (1996). Two types of network oscillations in neocortex mediated by distinct glutamate receptor subtypes and neuronal populations. J. Neurophysiol..

[207] Klimesch W. (2012). Alpha-band oscillations, attention, and controlled access to stored information. Trends Cogn. Sci..

[208] Alais D., Blake R. (2005). Binocular Rivalry.

[209] Blake R. (1989). A neural theory of binocular rivalry. Psychol. Rev..

[210] Wilson H.R. (2007). Minimal physiological conditions for binocular rivalry and rivalry memory. Vis. Res..

[211] Wilkinson F., Karanovic O., Wilson H. (2008). Binocular rivalry in migraine. Cephalalgia.

[212] McKendrick A.M., Battista J., Snyder J.S., Carter O.L. (2011). Visual and auditory perceptual rivalry in migraine. Cephalalgia.

[213] Robertson C.E., Ratai E.M., Kanwisher N. (2016). Reduced GABAergic action in the autistic brain. Curr. Biol..

[214] Van Loon A.M., Knapen T., Scholte H.S., John-Saaltink E.S., Donner T.H., Lamme V.A. (2013). GABA shapes the dynamics of bistable perception. Curr. Biol..

[215] Mentch J., Spiegel A., Ricciardi C., Robertson C.E. (2019). GABAergic inhibition gates perceptual awareness during binocular rivalry. J. Neurosci..

[216] Katyal S., He S., He B., Engel S.A. (2019). Frequency of alpha oscillation predicts individual differences in perceptual stability during binocular rivalry. Hum. Brain Mapp..

[217] Battista J., Badcock D.R., McKendrick A.M. (2011). Migraine increases centre-surround suppression for drifting visual stimuli. PLoS ONE.

[218] Field D.T., Cracknell R.O., Eastwood J.R., Scarfe P., Williams C.M., Zheng Y., Tavassoli T. (2022). High-dose Vitamin B6 supplementation reduces anxiety and strengthens visual surround suppression. Hum. Psychopharmacol. Clin. Exp..

[219] Martin D.L. (1994). Pyridoxal Phosphate, GABA and Seizure Susceptibility. Proceedings of the Biochemistry of Vitamin B 6 and PQQ.

[220] Bertalmío M., Calatroni L., Franceschi V., Franceschiello B., Gomez Villa A., Prandi D. (2020). Visual illusions via neural dynamics: Wilson–Cowan-type models and the efficient representation principle. J. Neurophysiol..

[221] Shepherd A. (2000). Visual contrast processing in migraine. Cephalalgia.

[222] Shepherd A.J. (2006). Local and global motion after-effects are both enhanced in migraine, and the underlying mechanisms differ across cortical areas. Brain.

[223] Tibber M.S., Guedes A., Shepherd A.J. (2006). Orientation discrimination and contrast detection thresholds in migraine for cardinal and oblique angles. Investig. Ophthalmol. Vis. Sci..

[224] Tibber M.S., Kelly M.G., Jansari A., Dakin S.C., Shepherd A.J. (2014). An inability to exclude visual noise in migraine. Investig. Ophthalmol. Vis. Sci..

[225] Krause M.R., Vieira P.G., Thivierge J.P., Pack C.C. (2022). Brain stimulation competes with ongoing oscillations for control of spike timing in the primate brain. PLoS Biol..

[226] Bigal M., Rapoport A., Aurora S., Sheftell F., Tepper S., Dahlof C. (2007). Satisfaction with current migraine therapy: Experience from 3 centers in US and Sweden. Headache J. Head Face Pain.

[227] Silberstein S.D. (2015). Preventive migraine treatment. Contin. Lifelong Learn. Neurol..

[228] Andreou A.P., Fuccaro M., Lambru G. (2020). The role of erenumab in the treatment of migraine. Ther. Adv. Neurol. Disord..

[229] Uddman R., Edvinsson L., Ekman R., Kingman T., McCulloch J. (1985). Innervation of the feline cerebral vasculature by nerve fibers containing calcitonin gene-related peptide: Trigeminal origin and co-existence with substance P. Neurosci. Lett..

[230] Iyengar S., Johnson K.W., Ossipov M.H., Aurora S.K. (2019). CGRP and the trigeminal system in migraine. Headache J. Head Face Pain.

[231] Edvinsson L. (2015). CGRP receptor antagonists and antibodies against CGRP and its receptor in migraine treatment. Br. J. Clin. Pharmacol..

[232] Martin D.L. (1987). Regulatory properties of brain glutamate decarboxylase. Cell. Mol. Neurobiol..

[233] Smith A.K., Wade A.R., Penkman K.E., Baker D.H. (2017). Dietary modulation of cortical excitation and inhibition. J. Psychopharmacol..

[234] Ikeda M., Azuma S., Inoué S. (1997). Vitamin B12 enhances GABA content but reduces glutamate content in the rat suprachiasmatic nucleus. Am. J. -Physiol.-Regul. Integr. Comp. Physiol..

[235] Chen Y.S., Lee H.F., Tsai C.H., Hsu Y.Y., Fang C.J., Chen C.J., Hung Y.H., Hu F.W. (2022). Effect of Vitamin B2 supplementation on migraine prophylaxis: A systematic review and meta-analysis. Nutr. Neurosci..

[236] Colombo B., Saraceno L., Comi G. (2014). Riboflavin and migraine: The bridge over troubled mitochondria. Neurol. Sci..

[237] Holton K.F. (2021). Micronutrients may Be a unique weapon against the neurotoxic triad of excitotoxicity, oxidative stress and neuroinflammation: A perspective. Front. Neurosci..

[238] Kuriemann G., Löscher W., Dominick H., Palm G. (1987). Disappearance of neonatal seizures and low CSF GABA levels after treatment with vitamin B6. Epilepsy Res..

[239] Rogawski M.A. (2012). Migraine and epilepsy—Shared mechanisms within the family of episodic disorders. Jasper’s Basic Mechanisms of the Epilepsies [Internet].

[240] Kretsch M.J., Sauberlich H.E., Newbrun E. (1991). Electroencephalographic changes and periodontal status during short-term vitamin B-6 depletion of young, nonpregnant women. Am. J. Clin. Nutr..

[241] Plecko B., Stöckler S. (2009). Vitamin B6 dependent seizures. Can. J. Neurol. Sci. J. Can. Des Sci. Neurol..

[242] Ghin F., O’Hare L., Pavan A. (2021). Electrophysiological aftereffects of high-frequency transcranial random noise stimulation (hf-tRNS): An EEG investigation. Exp. Brain Res..

[243] Yeh T.C., Huang C.C.Y., Chung Y.A., Im J.J., Lin Y.Y., Ma C.C., Tzeng N.S., Chang H.A. (2022). High-frequency transcranial random noise stimulation modulates gamma-band EEG source-based large-scale functional network connectivity in patients with schizophrenia: A randomized, double-blind, sham-controlled clinical trial. J. Pers. Med..

[244] Bhola R., Kinsella E., Giffin N., Lipscombe S., Ahmed F., Weatherall M., Goadsby P.J. (2015). Single-pulse transcranial magnetic stimulation (sTMS) for the acute treatment of migraine: Evaluation of outcome data for the UK post market pilot program. J. Headache Pain.

[245] O’Hare L., Griffiths R. (2022). Transcranial Electrical Stimulation in Migraine–How Does It Work and What Can We Learn from It?. OBM Neurobiol..

[246] Antal A., Kriener N., Lang N., Boros K., Paulus W. (2011). Cathodal transcranial direct current stimulation of the visual cortex in the prophylactic treatment of migraine. Cephalalgia.

[247] Mansour A.G., Ahdab R., Khazen G., El-Khoury C., Sabbouh T.M., Salem M., Yamak W., Chalah M.A., Ayache S.S., Riachi N. (2020). Transcranial direct current stimulation of the occipital cortex in medication overuse headache: A pilot randomized controlled cross-over study. J. Clin. Med..

[248] Auvichayapat P., Janyacharoen T., Rotenberg A., Tiamkao S., Krisanaprakornkit T., Sinawat S., Punjaruk W., Thinkhamrop B., Auvichayapat N. (2012). Migraine prophylaxis by anodal transcranial direct current stimulation, a randomized, placebo-controlled trial. J. Med. Assoc. Thai..

[249] Andrade S.M., de Brito Aranha R.E.L., de Oliveira E.A., de Mendonça C.T.P.L., Martins W.K.N., Alves N.T., Fernández-Calvo B. (2017). Transcranial direct current stimulation over the primary motor vs prefrontal cortex in refractory chronic migraine: A pilot randomized controlled trial. J. Neurol. Sci..

[250] Dalla Volta G., Marceglia S., Zavarise P., Antonaci F. (2020). Cathodal tDCS guided by thermography as adjunctive therapy in chronic migraine patients: A sham-controlled pilot study. Front. Neurol..

[251] Rahimi M.D., Fadardi J.S., Saeidi M., Bigdeli I., Kashiri R. (2020). Effectiveness of cathodal tDCS of the primary motor or sensory cortex in migraine: A randomized controlled trial. Brain Stimul..

[252] De Koninck B.P., Brazeau D., Guay S., Babiloni A.H., De Beaumont L. (2023). Transcranial Alternating Current Stimulation to Modulate Alpha Activity: A Systematic Review. Neuromodul. Technol. Neural Interface.

[253] Antal A., Bischoff R., Stephani C., Czesnik D., Klinker F., Timäus C., Chaieb L., Paulus W. (2020). Low intensity, transcranial, alternating current stimulation reduces migraine attack burden in a home application set-up: A double-blinded, randomized feasibility study. Brain Sci..

[254] Ward L.M. (2009). Physics of neural synchronisation mediated by stochastic resonance. Contemp. Phys..

[255] Pavan A., Ghin F., Contillo A., Milesi C., Campana G., Mather G. (2019). Modulatory mechanisms underlying high-frequency transcranial random noise stimulation (hf-tRNS): A combined stochastic resonance and equivalent noise approach. Brain Stimul..

[256] O’Hare L., Goodwin P., Sharp A., Contillo A., Pavan A. (2021). Improvement in visual perception after high-frequency transcranial random noise stimulation (hf-tRNS) in those with migraine: An equivalent noise approach. Neuropsychologia.

[257] Garg S., Williams S., Jung J., Pobric G., Nandi T., Lim B., Vassallo G., Green J., Evans D.G., Stagg C.J. (2022). Non-invasive brain stimulation modulates GABAergic activity in Neurofibromatosis 1. Sci. Rep..

[258] Bachtiar V., Near J., Johansen-Berg H., Stagg C.J. (2015). Modulation of GABA and resting state functional connectivity by transcranial direct current stimulation. Elife.

[259] Benwell C.S., Coldea A., Harvey M., Thut G. (2022). Low pre-stimulus EEG alpha power amplifies visual awareness but not visual sensitivity. Eur. J. Neurosci..

[260] Iemi L., Busch N.A., Laudini A., Haegens S., Samaha J., Villringer A., Nikulin V.V. (2019). Multiple mechanisms link prestimulus neural oscillations to sensory responses. Elife.

[261] Bastos A.M., Lundqvist M., Waite A.S., Kopell N., Miller E.K. (2020). Layer and rhythm specificity for predictive routing. Proc. Natl. Acad. Sci. USA.

[262] Kitzbichler M.G., Khan S., Ganesan S., Vangel M.G., Herbert M.R., Hämäläinen M.S., Kenet T. (2015). Altered development and multifaceted band-specific abnormalities of resting state networks in autism. Biol. Psychiatry.

[263] Tarasi L., Magosso E., Ricci G., Ursino M., Romei V. (2021). The directionality of fronto-posterior brain connectivity is associated with the degree of individual autistic traits. Brain Sci..

[264] Wang J., Barstein J., Ethridge L.E., Mosconi M.W., Takarae Y., Sweeney J.A. (2013). Resting state EEG abnormalities in autism spectrum disorders. J. Neurodev. Disord..

[265] Ursino M., Serra M., Tarasi L., Ricci G., Magosso E., Romei V. (2022). Bottom-up vs. top-down connectivity imbalance in individuals with high-autistic traits: An electroencephalographic study. Front. Syst. Neurosci..

[266] Tarasi L., Borgomaneri S., Romei V. (2023). Antivax attitude in the general population along the autism-schizophrenia continuum and the impact of socio-demographic factors. Front. Psychol..

[267] Sullivan J.C., Miller L.J., Nielsen D.M., Schoen S.A. (2014). The presence of migraines and its association with sensory hyperreactivity and anxiety symptomatology in children with autism spectrum disorder. Autism.

[268] Webster K.E., Dickinson J.E., Battista J., McKendrick A.M., Badcock D.R. (2012). Evidence for increased internal noise in migraineurs for contrast and shape processing. Cephalalgia.

[269] Trajkovic J., Di Gregorio F., Avenanti A., Thut G., Romei V. (2023). Two oscillatory correlates of attention control in the alpha-band with distinct consequences on perceptual gain and metacognition. J. Neurosci..

[270] Coldea A., Veniero D., Morand S., Trajkovic J., Romei V., Harvey M., Thut G. (2022). Effects of Rhythmic Transcranial Magnetic Stimulation in the Alpha-Band on Visual Perception Depend on Deviation From Alpha-Peak Frequency: Faster Relative Transcranial Magnetic Stimulation Alpha-Pace Improves Performance. Front. Neurosci..

[271] Ronconi L., Busch N.A., Melcher D. (2018). Alpha-band sensory entrainment alters the duration of temporal windows in visual perception. Sci. Rep..

[272] Wutz A., Melcher D., Samaha J. (2018). Frequency modulation of neural oscillations according to visual task demands. Proc. Natl. Acad. Sci. USA.

[273] Cooke J., Poch C., Gillmeister H., Costantini M., Romei V. (2019). Oscillatory properties of functional connections between sensory areas mediate cross-modal illusory perception. J. Neurosci..

